# 8.2% of the Human Genome Is Constrained: Variation in Rates of Turnover across Functional Element Classes in the Human Lineage

**DOI:** 10.1371/journal.pgen.1004525

**Published:** 2014-07-24

**Authors:** Chris M. Rands, Stephen Meader, Chris P. Ponting, Gerton Lunter

**Affiliations:** 1 MRC Functional Genomics Unit, Department of Physiology, Anatomy, and Genetics, University of Oxford, Oxford, United Kingdom; 2 Wellcome Trust Centre for Human Genetics, University of Oxford, Oxford, United Kingdom; Aarhus University, Denmark

## Abstract

Ten years on from the finishing of the human reference genome sequence, it remains unclear what fraction of the human genome confers function, where this sequence resides, and how much is shared with other mammalian species. When addressing these questions, functional sequence has often been equated with pan-mammalian conserved sequence. However, functional elements that are short-lived, including those contributing to species-specific biology, will not leave a footprint of long-lasting negative selection. Here, we address these issues by identifying and characterising sequence that has been constrained with respect to insertions and deletions for pairs of eutherian genomes over a range of divergences. Within noncoding sequence, we find increasing amounts of mutually constrained sequence as species pairs become more closely related, indicating that noncoding constrained sequence turns over rapidly. We estimate that half of present-day noncoding constrained sequence has been gained or lost in approximately the last 130 million years (half-life in units of divergence time, *d_1/2_* = 0.25–0.31). While enriched with ENCODE biochemical annotations, much of the short-lived constrained sequences we identify are not detected by models optimized for wider pan-mammalian conservation. Constrained DNase 1 hypersensitivity sites, promoters and untranslated regions have been more evolutionarily stable than long noncoding RNA loci which have turned over especially rapidly. By contrast, protein coding sequence has been highly stable, with an estimated half-life of over a billion years (*d_1/2_* = 2.1–5.0). From extrapolations we estimate that 8.2% (7.1–9.2%) of the human genome is presently subject to negative selection and thus is likely to be functional, while only 2.2% has maintained constraint in both human and mouse since these species diverged. These results reveal that the evolutionary history of the human genome has been highly dynamic, particularly for its noncoding yet biologically functional fraction.

## Author Summary

Nearly 99% of the human genome does not encode proteins, and while there recently has been extensive biochemical annotation of the remaining noncoding fraction, it remains unclear whether or not the bulk of these DNA sequences have important functional roles. By comparing the genome sequences of different species we identify genomic regions that have evolved unexpectedly slowly, a signature of natural selection upon functional sequence. Using a high resolution evolutionary approach to find sequence showing evolutionary signatures of functionality we estimate that a total of 8.2% (7.1–9.2%) of the human genome is presently functional, more than three times as much than is functional and shared between human and mouse. This implies that there is an abundance of sequences with short lived lineage-specific functionality. As expected, most of the sequence involved in this functional “turnover” is noncoding, while protein coding sequence is stably preserved over longer evolutionary timescales. More generally, we find that the rate of functional turnover varies significantly across categories of functional noncoding elements. Our results provide a pan-mammalian and whole genome perspective on how rapidly different classes of sequence have gained and lost functionality down the human lineage.

## Introduction

“What proportion of the human genome is functional?” remains a contentious question [1]–[3]. In great part this reflects the use of definitions of ‘function’ that differ from the traditional definition that is based on fitness and selection (see e.g. [4] for a discussion). For instance, equating functionality with annotation by at least one of the ENCODE consortium's biochemical assays [5] results in approximately 80% of the human genome being labeled as functional [1], [6]. While this approach has the advantage of being empirical, it makes the definition of functionality dependent on the choice of experiments and details such as P value cutoffs. It is also questionable whether, for instance, introns should be classified as functional based merely on their transcription [2], [4].

By contrast, evolutionary studies often equate functionality with signatures of selection. While it is undisputed that many functional regions have evolved under complex selective regimes including selective sweeps [7] or ongoing balancing selection [8], [9], and it appears likely that loci exist where recent positive selection or reduction of constraint has decoupled deep evolutionary patterns from present functional status [10], [11], it is widely accepted that purifying selection persisting over long evolutionary times is a ubiquitous mode of evolution [12], [13]. While acknowledging the caveats, this justifies the definition of functional nucleotides used here, as those that are *presently* subject to *purifying* selection.

This is of course not useful as an operational definition, as selection cannot be measured instantaneously. Instead, most studies define functional sites as those subject to purifying selection between two (or more) particular species. Studies that follow this definition have estimated the proportion of functional nucleotides in the human genome, denoted as α_sel_ [14], [15], between 3% and 15% ([3] and references therein, [16]). Since each species' lineage gains and loses functional elements over time, α_sel_ needs to be understood in the context of divergence between species. The divergence influences the estimate of α_sel_ in two ways. On the one hand, constrained sequence between closely related species, including lineage-specific constrained sequence, is harder to detect than more broadly conserved sequence because of a paucity of informative mutations, which reduces detection power. On the other hand, estimates of constraint between any two species will only include sequence that was present in their common ancestor and that has been constrained in the lineages leading up to both extant species' genomes, with the consequence that turnover of functional sequence leads to diminishing α_sel_ estimates as the species divergence increases. Assuming that the first effect can be controlled for, higher estimates of sequence constraint that are obtained between more closely related species [15], [17] are thus indicative of the turnover of functional sequence [15]. Here we understand turnover to mean the loss or gain of purifying selection at a particular locus of the genome, when changes in the physical or genetic environment, or mutations at the locus itself, cause the locus to switch from being functional to being non-functional or vice versa.

Two previous studies have made quantitative estimates of the overall rate of turnover ([15], [17], reviewed in [3]). The estimate by Smith *et al.* (2004) [17] was derived from an analysis of point mutations in alignments across a 1.8 Mb genomic region. While a high rate of turnover was inferred, the authors emphasised the preliminary nature of their work as a consequence of the limited amount of data available to them at that time. Later, Meader *et al.* (2010) [15] performed genome-wide analysis with a neutral indel model (see [18], here referred to as NIM1) to estimate the fraction, termed α_selIndel_, of human sequence that was constrained with respect to insertions or deletion mutations (indels). This study also found a high rate of turnover, and estimated using two *ad hoc* heuristic approaches that 6.5–10% of the human genome is functional. Extrapolations using these data subsequently suggested that 10–15% of the human genome is presently functional [3].

NIM1 is a quantitative model describing the distribution of distances between neighbouring indels (intergap segments; IGSs) in neutrally evolving sequence, which provides an excellent description of the observed frequency of medium-sized IGSs. However, across whole genome alignments longer IGSs are strikingly overrepresented compared to this expectation under neutrality, presumably as a result of the presence of functional genomic segments under purifying selection in which indel mutations are unlikely to become fixed. By quantifying this overrepresentation it is possible to estimate α_selIndel_, the fraction of nucleotides contained within these functional segments. The model (which also accounts for G+C content and sex chromosome-dependent mutational biases) performs well for simulated data, and accurately identifies coding regions and ancestral repeats as highly conserved and neutrally evolving, respectively [15], [18]. However, some concerns about the model's derivation and the quality of whole-genome alignments we used were subsequently brought to our attention, which motivated us to initiate this study.

Here we present improved methods for the estimation of α_selIndel_ and the inference of functional turnover, building on our previous approaches [15], [18]. We apply these improved approaches to pairwise alignments between the genomes of diverse eutherian mammals, and we estimate that 7.1–9.2% of the human genome is presently subject to purifying selection, equating to 220–286 Mb of constrained sequence. We also take advantage of the additional high-quality eutherian genome sequences that have become available since our previous study to provide improved estimates of the rate of turnover of functional sequence in these species. Improvements in biological and biochemical annotation of genomic sequence mean that we can investigate turnover rates within particular classes of functional elements, such as coding sequences, DNase 1 hypersensitivity sites (DNase HSs), transcription factor binding sites (TFBSs), enhancers, promoters, and long noncoding RNAs (lncRNAs). We find striking differences between the functional element classes; in particular constrained coding sequences are much more evolutionary stable than constrained noncoding sequences, and lncRNAs show the most rapid rate of turnover of all the noncoding element types.

## Results

**Figure 1:**
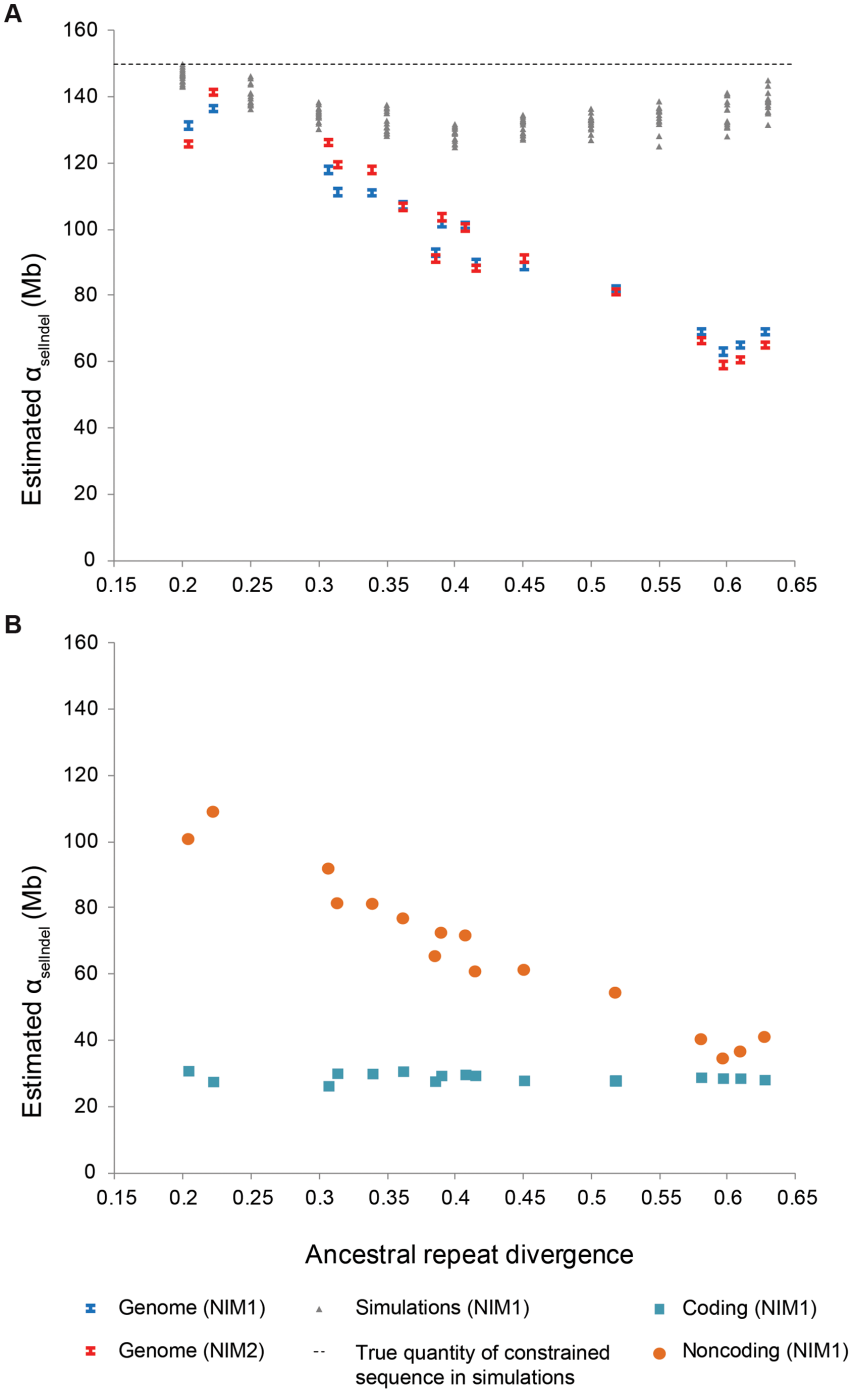
Evolutionary turnover of constrained sequence. A. Quantity of constrained sequence (α_selIndel_) estimated by NIM1 (blue bars) and NIM2 (red bars) plotted against ancestral repeat divergence for different pairs of eutherian species genomes, with the simulated data (grey) shown under a non-turnover scenario. B. Coding sequence (blue squares) is seen to be broadly conserved, while constrained noncoding sequence (orange circles) shows a strong negative correlation between α_selIndel_ and divergence, indicating rapid turnover.

We developed three improvements for estimating α_selIndel_. First, we identified two issues in the original derivation of the NIM1 model, and found that corrections result in equal but opposite changes in the inferred α_selIndel_, so that these issues do not invalidate the original results (Text S1). To provide further assurance of the accuracy of the derivation we introduced a new likelihood neutral indel model (NIM2) that provides a partially independent validation of the revised NIM1 estimates (Text S2). Second, we find that earlier α_selIndel_ estimates were upwardly biased as a consequence of poor quality alignments (Materials and Methods; Text S3; Figure S1; Figure S2). Third, we significantly extended the original simulation study, testing the influence of a wide range of modelling assumptions on the inferences. Results underscored the validity, accuracy and robustness of the model (Text S4; Text S5; Figure 1A; Figure S3).

We applied the neutral indel model to estimate α_selIndel_ on trimmed whole genome alignments between a wide range of eutherian species pairs for which high quality genome assemblies are available. Estimates of α_selIndel_ (blue symbols in Figure 1A; Table 1) were largely concordant with the likelihood neutral indel model NIM2 (red symbols). Our new estimates are considerably reduced (by 10%–40%) compared to our previous α_selIndel_ estimates (Table 1) [15]. These differences are largely attributable to alignment trimming. Previously we reported lower and higher bounds for α_selIndel_ under two assumptions of the extent of clustering of functional sequence [15], but simulations indicate that the higher bound is irrelevant under all but unrealistically strong clustering. We therefore now report the lower bound only, and in addition provide 95% confidence intervals obtained from regression estimates and standard assumptions on error distributions. (Materials and Methods; Text S3).

**Table 1:** Estimated quantities of sequence constrained with respect to indels (α_selIndel_) between different species under different models.

Species pair	Estimated quantity of constrained sequence (Mb)			Ancestral repeat divergence
	NIM1	NIM2	Meader *et al.* 2010	
Human – Horse	110.5–112.0	118.9–120.1	150.8–200.8	0.339
Human – Rhino	110.8–112.1	119.7–120.9	N/A	0.313
Human – Bushbaby	106.8–108.2	109.1–110.2	N/A	0.362
Human – Dog	100.8–101.9	101.7–102.6	121.8–151.1	0.407
Human – Panda	101.4–102.5	105.5–106.4	N/A	0.390
Human – Cattle	89.8–90.6	90.7–91.6	114.3–143.6	0.415
Human – Rabbit	88.8–89.7	93.0–93.9	N/A	0.450
Human – Guinea pig	81.9–82.7	81.2–82.0	N/A	0.517
Human – Mouse	68.8–69.4	66.6–67.1	81.4–96.2	0.627
Mouse – Rat	130.4–132.9	125.6–127.5	189.0–258.4	0.204
Mouse – Horse	68.9–69.5	66.5–67.1	76.3–91.0	0.580
Mouse – Dog	64.9–65.5	60.8–61.3	71.1–83.0	0.609
Mouse – Cattle	62.9–63.4	56.4–56.9	63.8–74.5	0.596
Dog – Ferret	135.6–137.7	141.2–142.9	N/A	0.222
Dog – Horse	117.4–118.9	126.5–127.8	147.6–194.5	0.307
Dog – Cattle	92.5–93.6	91.3–92.2	114.8–144.0	0.385

There is good agreement between the estimates inferred by NIM1 and NIM2, but previous estimates of [15] are considerably higher, mainly owing to alignment artefacts.

### Rapid turnover of functional sequence across eutherian evolution

We observe a strong negative correlation between estimates of α_selIndel_ and the divergence of the two species being compared (Figure 1), consistent with substantial turnover of functional sequence and thus with earlier conclusions [15], [17], and inconsistent with simulation results under a scenario in which turnover is absent (Figure 1A).

**Figure 2:**
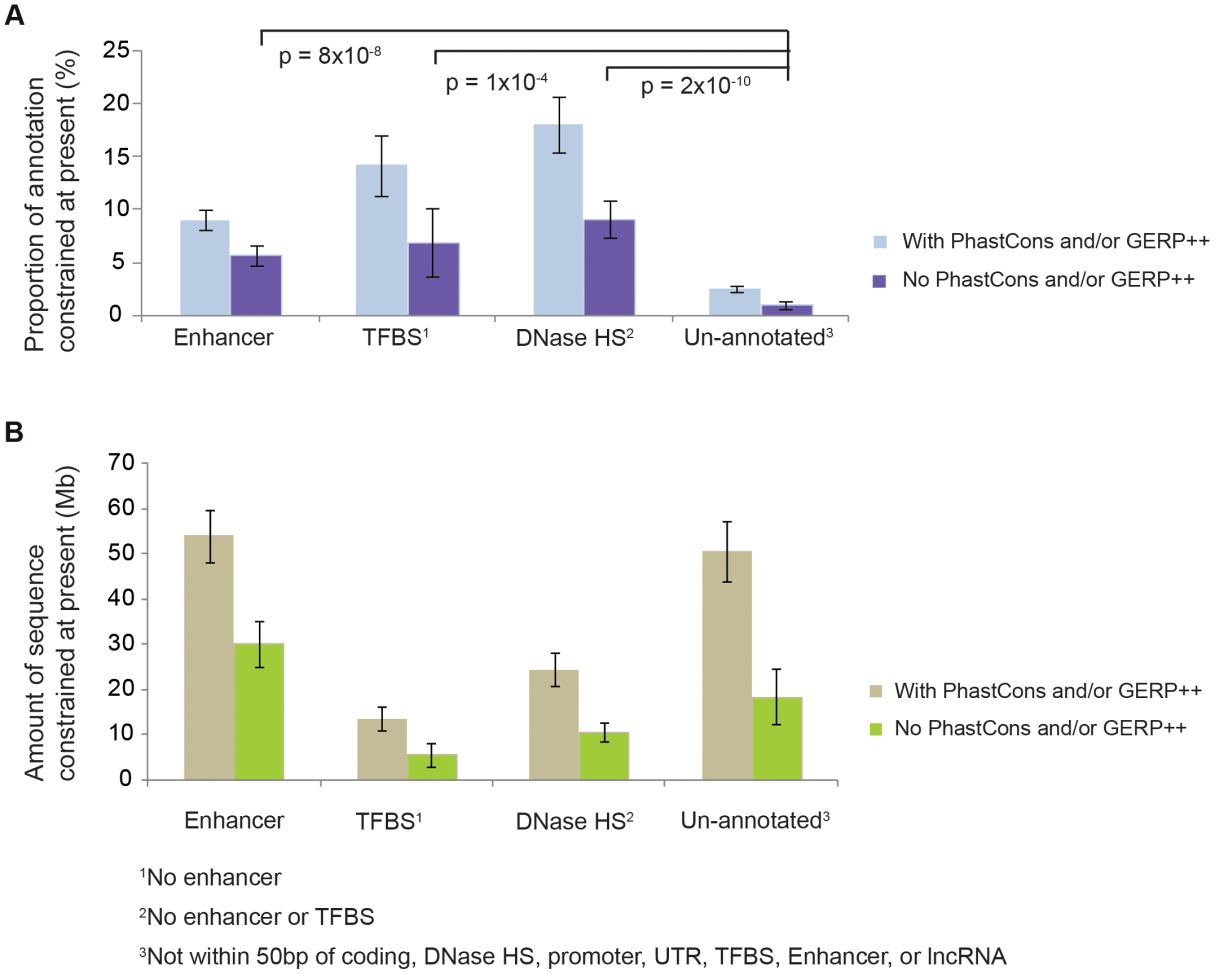
The overlap of constrained sequence with pan-mammalian conserved sequences. The proportions A., and quantities B., of constrained sequence at the present for different types of biochemically annotated and un-annotated sequences, with and without PhastCons or GERP++ conserved elements, estimated using linear extrapolations (Text S6, Text S7). The NIM1 has power to detect functional lineage-specific constrained sequence: NIM1 detects significantly higher fractions of linage-specific constrained sequence (defined as sequence identified by NIM1 but not annotated by PhastCons or GERP++ as being conserved across mammals) within 3 mutually exclusive classes of ENCODE biochemical annotations compared to sequence lacking such annotation; see Text S6 for details.

To exclude the possibility that technical artefacts are driving this observation, we investigated ENCODE annotations in lineage-specific NIM1-constrained sequence. Specifically, we identified NIM1-constrained sequence that was not identified as pan-mammalian conserved by either the PhastCons [12] or GERP++ algorithms [19], and found that such sequence is enriched for biochemically annotated sequences (DNase HSs, TFBSs, and enhancers defined by the ENCODE consortium [5]) (Figure 2; Figure S4). This is expected if functional elements, including these ENCODE functional classes, have been subject to evolutionary turnover, but is not expected if technical artefacts were causing the observations in Figure 1. Furthermore, using low-frequency polymorphic indels from the 1000 Genomes project we could exclude the possibility that lower mutation rates in ENCODE functional regions were causing the observations. We therefore conclude that observations in Figure 1 reflect turnover of functional elements. A more detailed discussion on this issue is provided in Text S6 and Text S7.

### A model for sequence turnover

To help describe and interpret the observations of turnover (Figure 1) we propose a time-homogeneous model for sequence turnover on a genomic scale. We apply this model to specific sequence classes, such as protein coding genes or TFBSs, allowing us to discuss the rates of turnover for particular types of functional element. The model assumes that within a particular functional class both the total amount *a* of functional sequence and the rate *b* of turnover per nucleotide (nt) are constant, and that the turnover rate is the same for all nts in a class. Under this model the total amount of functional nts in any class remains constant over time, but the amount that is currently functional and retains homology to functional nts in the ancestral species at divergence *d* (i.e., the amount that was constrained and has not turned over in the course of evolution to the present) is 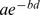. We estimate the parameters *a* and *b* by fitting the model to observations using weighted linear regression (Materials and Methods). Instead of the rate parameter *b*, we, equivalently, often refer to the *turnover half life*, *d_1/2_*, which is defined as the divergence at which half the functional sequences in the class is expected to have turned over and is calculated as log_e_(2)/*b*. We express this divergence in time units corresponding to one expected nucleotide substitution per site in neutrally evolving sequence (‘divergence time’). To convert this divergence to years, we apply a substitution rate of 2.2×10^−9^ per site per year [20]. This will be a more appropriate value for the human lineage, on which we focus, than on rodent lineages whose per-year substitution rate are substantially higher.

The model is time-reversible, so that the same expression describes the amount of mutually constrained sequence between two extant species at divergence *d*, where *d* is calculated by adding the divergences along the two branches to their last common ancestor. Similarly, to convert *d* (in years) to the age of the most recent common ancestor, it should be divided by 2.

To calculate the divergence time we use ancestral repeats (ARs, sequence derived from transposable elements whose insertion predates the species' last common ancestor) as a proxy for neutrally evolving sequence, because they virtually all show the patterns of indel mutation expected under neutral evolution [18]. Our estimates of divergence using either ARs or synonymous sites as neutral proxy are concordant, hence our results are insensitive to the choice of putatively neutral sequence (Figure S5).

### Different turnover rates for coding and noncoding sequence

We next used NIM1 to estimate the fraction of constrained sequence within coding and noncoding sequences (Materials and Methods). Within protein coding sequence selective constraint is pervasive, as expected (Figure 1B): 80–88% of human or mouse annotated coding sequence has been under selective constraint with respect to indels across eutherian evolution; slightly lower proportions were estimated under the NIM2 and for dog annotated coding sequences (Figure S6; Text S8).

In contrast to protein coding sequence, estimates for the extent of constraint in noncoding sequence show a pronounced drop-off with increasing divergence (orange filled circles in Figure 1B), an observation compatible with turnover occurring predominantly within the noncoding functional fraction of the genome. When applying the time-homogeneous turnover model to these data, we estimate the turnover rate parameter *b* for noncoding sequence at 2.48 turnover events per neutral substitution (2.26–2.71, 95% confidence interval), equivalent to a turnover half life *d_1/2_* of 0.28 (0.25–0.31) in units of divergence time, or 127 My (116–139 My) in natural time units. The present estimate represents a slower turnover rate than a previous estimate of *d_1/2_* = 0.19 (86 My) made by Ponting *et al.* (2011) [3] with data from Meader *et al.* (2010) [15].

We observe a low yet significantly non-zero rate of turnover in coding sequence, *b* = 0.24 (0.14–0.33) events per neutral substitution, corresponding to *d_1/2_* = 2.9 (2.1–5.0), or in natural units 1300 My (950–2250 My). These estimates represent an average across the undoubtedly variable rates of turnover across different types of protein coding gene sequence. Nevertheless, under this simple model, we find that protein coding sequence is relatively evolutionarily stable, showing long-term conservation, so that assuming that protein coding sequences exhibit no turnover will often be justified (e.g. [3]). By contrast, present-day constrained noncoding sequence is less stable, being relatively rapidly gained and lost in a lineage-specific manner.

### Constraint and turnover among classes of human constrained element

**Figure 3:**
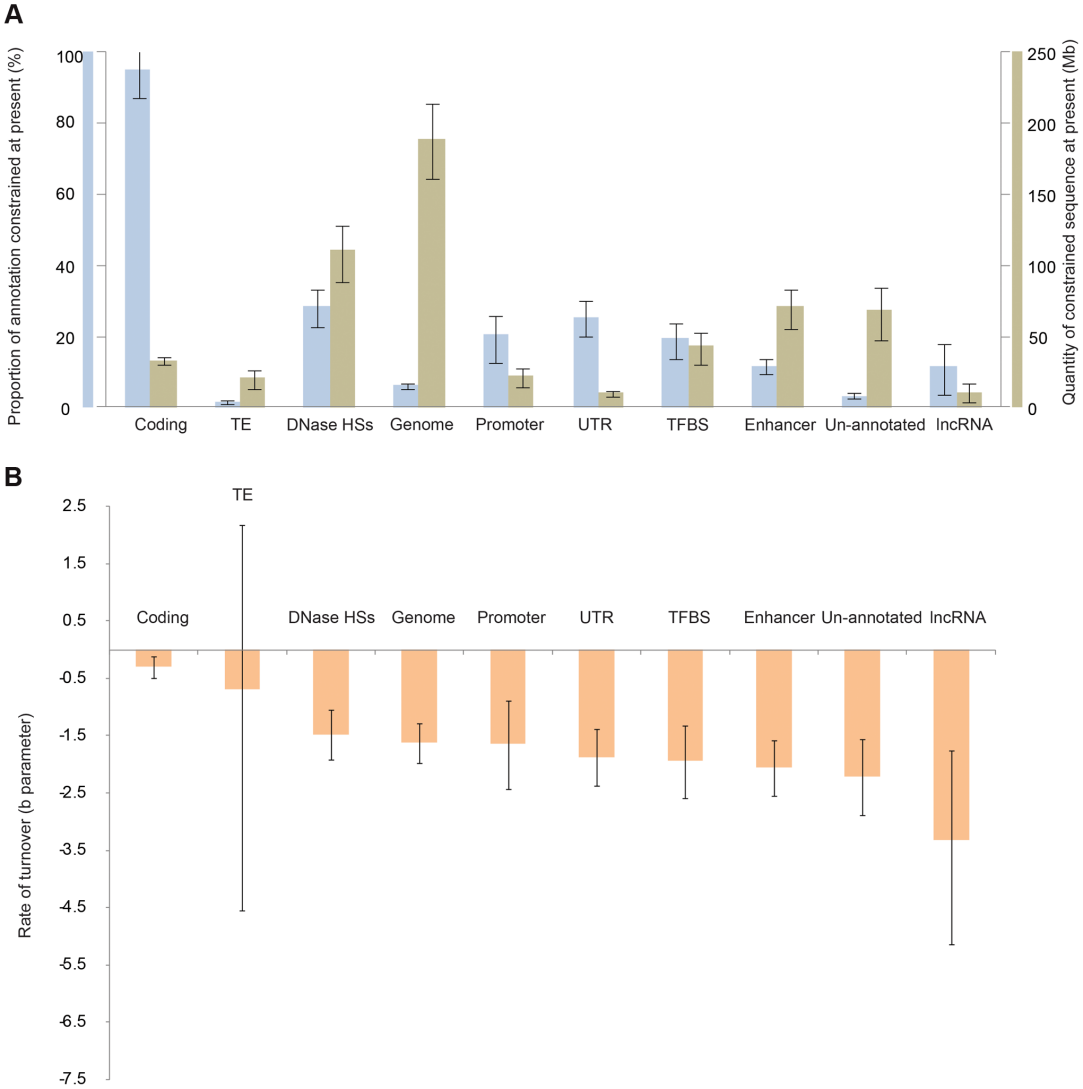
Constraint and turnover for different classes of human functional element. A. The total quantities of constrained sequence estimated for the present day by extrapolation for different element types. B. The estimated rate of turnover (b parameter) for different types of constrained element.

We next investigated whether various classes of functional element, identified in human primarily by the ENCODE project [5], exhibit contrasting levels of constraint, and whether these constrained element classes show a propensity to turn over at different rates. Of the functional classes we considered, promoters, untranslated regions (UTRs), DNAse HSs and TFBSs, enhancers and un-annotated sequences (defined as sequences not within 50 bp of ENCODE DNAse HSs, TFBS loci, lncRNAs from [21], Ensembl coding sequence, or UTRs) all show intermediate levels of turnover (Figure 3; Figure S7, Figure S8). LncRNA sequences show the highest level of turnover (Figure 3; Figure S8), and an even higher rate of turnover was inferred when the ENCODE-defined lncRNAs were used rather than the set from [21] (Figure S9). The fraction of sequence that the model inferred to be under present day constraint also varied across these categories, with intermediate fractions inferred for UTRs, DNAse HSs and TFBSs, and lower fractions for lncRNAs and enhancers. As expected, the lowest fractions were observed for un-annotated sequence; nevertheless, in absolute terms the amount of constrained sequence in this category is considerable (70 Mb, 45–85 Mb) (Figure 3). Constrained sequence in this category may represent lineage-specific functional sequences that were not identified by the ENCODE project, for instance because of their function in tissues or developmental stages not investigated by ENCODE. Finally, transposable element-derived sequences show very small amounts of constraint, and as a result our methods have little power to detect turnover in this class.

### Distribution of functional classes in present-day functional DNA

**Figure 4:**
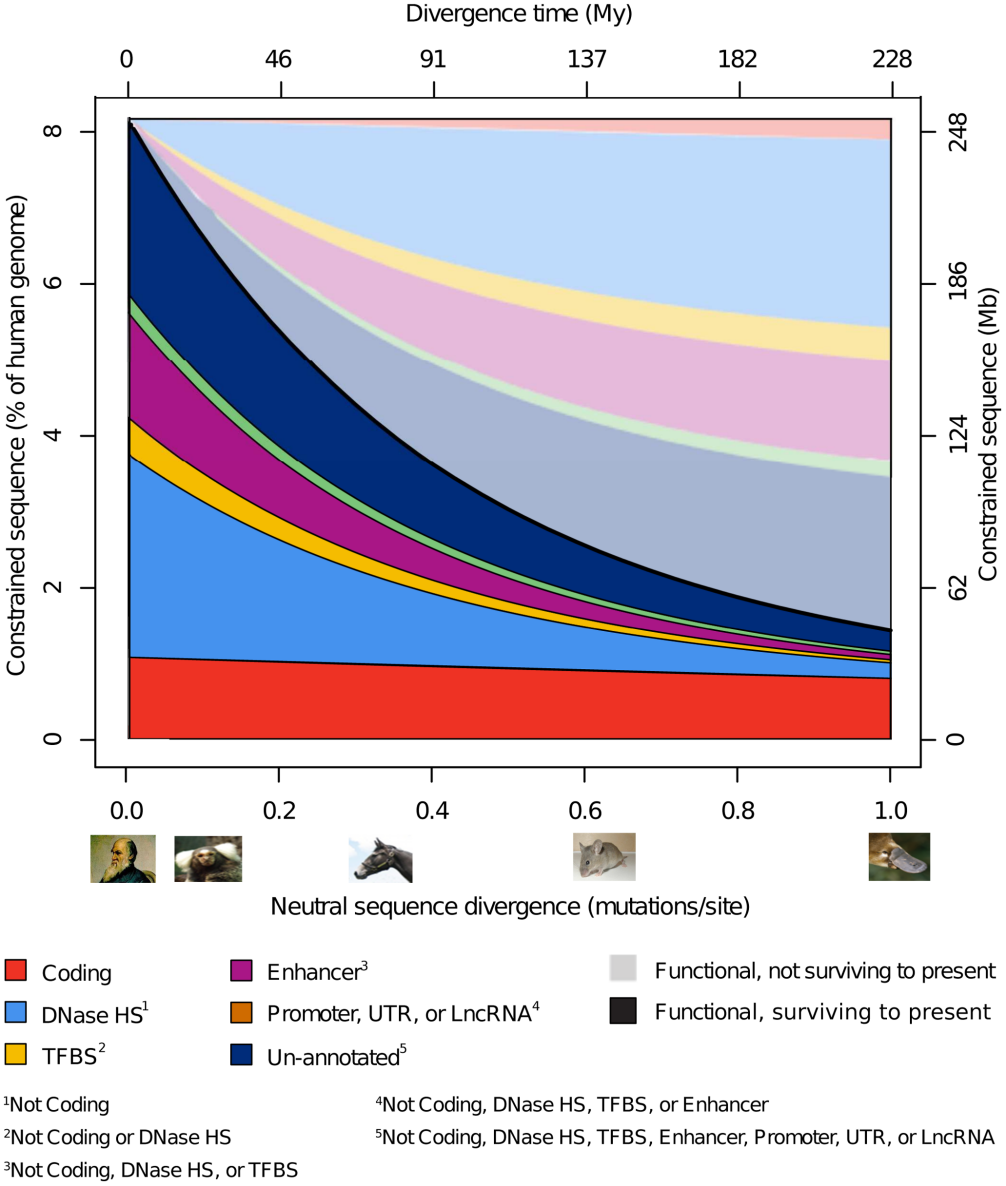
Model-based inference of turnover by functional class. Schematic summary of the fraction of constrained sequence that has been retained (saturated colours) or turned over (pastel colours) in the human lineage over time (X-axis, divergence time) and how it has been distributed across various categories of functional element. In addition to showing the reduced quantity of preserved constrained sequence with increasing divergence, we infer the reciprocal quantity of sequence that is assumed to have been gained over human lineage evolution. For consistency this approach requires mutually exclusive annotation sets, in contrast to those used in Figure 3, making the results not directly comparable. Overlaps between the major different annotations are shown in Figure S10.

We next examined how constrained sequence in the human genome is distributed cumulatively for selected functional element categories. We do this by fitting the functional turnover model to the observed data and extrapolating to the present day. In this way we also infer the reciprocal quantities of sequence that, when comparing to another species or human ancestor at a particular divergence, are presently functional in human yet have lost (or not gained) constraint in the lineage leading to the ancestor or other species (Figure 4). We stress that this inference relies on the parsimonious yet not formally justified assumption that the total quantity of functional sequence in genomes remains constant over time and therefore across species, and within functional categories. With these caveats we estimate that 8.6 Mb (26%) of constrained coding sequence has lost constraint (and thus has turned over) since the divergence of humans from monotremes approximately 228 million year ago (AR divergence time 1.00), while 200 Mb (79%) of the constrained noncoding human genome is inferred to have lost constraint over the same period. DNAse HSs cover more indel constrained sequence at all divergence ranges than all other annotated noncoding sequence combined, implying that DNAse HSs are an abundant and informative biochemical marker of functionality outside protein coding regions. Enhancers also show a marked contribution towards the constrained human genome, while TFBSs, promoters, UTRs and lncRNAs contribute considerably less sequence once their overlap with other annotations is removed. Finally, about a quarter of sequence inferred to be presently under constraint is not present in any of the annotation categories we considered. In Figure 4 we sum up the quantities of constrained sequence estimated from independent NIM1 runs for different annotation types.

### 7.1–9.2% of human genomes is constrained at present

If we make the assumption that the exponential decay model of functional sequence applies outside of the range of divergences we examined, then by extrapolating back to zero divergence we can estimate the total proportion of human genomes that is under present-day purifying selection with respect to indels. We perform this extrapolation across different annotation sets (Table S6). Although there is some variation in these estimates, we quote the estimate derived separately across multiple different annotation categories, namely coding sequence, DNase HSs, TFBS, Enhancers, unannotated sequence, and other sequence (the latter consisting of promoter, UTR and lncRNA sequences). This is because this estimate allows the rate of turnover to vary across each annotation type, and thus is likely to be more accurate than the estimates that assume a single rate of turnover across the whole genome, or the whole noncoding genome. We therefore estimate that 8.2% of the human genome (253 Mb; 95% CI 7.1%–9.2%, 220–286 Mb) is presently under purifying selection with respect to indels.

## Discussion

The question of what fraction of the human genome sequence are mutations preferentially purged owing to their deleterious effect has remained contentious ever since the first estimate was made in 2002 [22]. At that time it was not well appreciated that the amount of human constrained sequence that is also constrained in mouse is a minority (69 Mb; this study) of all human constrained sequence, owing to the relatively rapid gain and loss of functional sequence in their two lineages since their last common ancestor.

We find that NIM1-constrained sequence lacking evidence for pan-mammalian conservation is enriched for sequences with experimental evidence for biochemical activity, and we provide a detailed argument indicating that this is incompatible with the notion of technical artefacts causing the observed signature of turnover (Text S6). Extensive simulations indicating that estimates of constrained sequence are consistent across the divergence range we investigate further support this conclusion. Our estimate that 7.1–9.2% of human genomes is subject to contemporaneous selective constraint considerably exceeds previous estimates and falls short of others [3], [23]. We have shown that our method's previous estimates for specific species pairs, as well as the calculation that suggested 10–15% of the human genome is currently under negative selection were inflated [3], in large part owing to inaccuracies in whole genome alignments upon which our estimates were based. The problems associated with using whole-genome alignments could be circumvented entirely by instead using polymorphism data within a single species. However, this approach is technically highly challenging, and results have so far been controversial [16], [24], [25]; in addition this approach is not informative about functional turnover. Other published estimates [12], [18], [26] are lower because they, by design, were not sensitive to lineage-specific constrained sequence.

Our current estimates have their own particular caveats. While our results show that turnover is a real and substantial effect, simulations show that NIM1 underestimates the true amount of mutually constrained sequence to an extent that shows some dependence on the divergence. While simulations and theory indicate that point estimates of constraint remain conservative, the possibility of an upward bias in the inferred rate of *turnover* cannot be excluded, which in turn could lead to upwardly biased extrapolations of present-day constraint. In addition, the assumptions of the turnover model, in particular that all elements within a class are subject to the same rate of turnover, clearly are only approximately valid. These potential sources of error are not reflected in our confidence estimates (Table S6).

Our estimate that 7.1%–9.2% of the human genome is functional is around ten-fold lower than the quantity of sequence covered by the ENCODE defined elements [1], [5], [6]. This indicates that a large fraction of the sequence comprised by elements identified by ENCODE as having biochemical activity can be deleted without impacting on fitness. By contrast, the fraction of the human genome that is covered by coding exons, bound motifs and DNase1 footprints, all elements that are likely to contain a high fraction of nucleotides under selection, is 9%. While not all of the elements in these categories will be functional, and functional elements will exist outside of these categories, this figure is consistent with the proportion of sequence we estimate as being currently under the influence of selection.

As expected, turnover has occurred least in protein coding sequence, and thus has been most concentrated on noncoding sequence (Figure 4). For example, of the 43.5 Mb of sequence annotated by the ENCODE project as being within a human TFBS peak and that we find to be constrained (19.3% of the total extent of ENCODE TFBS peaks), only a third (30.6%; 13.3 Mb) is identified by NIM1 as being constrained in both human and mouse. A slightly higher proportion (45.6%; 19.8 Mb) is constrained in human and dog, presumably reflecting these species' lower divergence. These estimates are in good agreement with previous experimental findings: for instance 23–41% of TF binding events have been found to be conserved across human, dog and mouse for four liver TFs [27], while for two additional liver TFs, 7–14% of TF binding events are shared between human and mouse, and 15–20% between human and dog [28]. The phenomenon of turnover is well supported by both anecdotal evidence [27]–[29] and by broader studies of particular classes of elements, mostly TFBSs and enhancer elements [30]–[32]. The class of functional element inferred to turnover fastest was that of lncRNAs, again consistent with observations that most human lncRNAs are primate-specific and only 19% of lncRNAs are conserved over more than 90 My [33].

What our approach cannot clarify is to what extent the observed turnover at the sequence level amounts to different sequences encoding equivalent function [29], [30], or species-specific functional change [16], [31], [34]. Several lines of evidence, both from anecdotal [29] and broader [30], [31] studies of TFBSs, indicate that a large fraction of sequence changes involving TFBSs preserve function. For example, some deeply conserved transcription factors have species-specific binding sites in the vicinity of orthologous genes [27], [28] implying that despite their sequence divergence, the different DNA binding sites confer equivalent functions (on orthologous genes) in different lineages. Comprehensive studies of human and mouse embryonic heart enhancers found these to be weakly conserved [35], [36], despite human enhancers sequences largely driving expected tissue-specific expression in mouse embryonic heart tissue [36]. Another study found that two mammalian hypothalamic enhancers have no homolog across non-mammalian vertebrates, yet are still able to drive specific expression patterns in zebrafish neurons [37]. These findings are consistent with gene expression evolution being shaped predominantly by stabilizing selection on the expression level [38], while evolution on the sequence level may involve an interplay between fixation of weakly deleterious mutations through drift, and weak positive selection on compensatory mutations [39].

However, not all TFBS turnover events are neutral or nearly neutral on the level of gene expression, and the fraction of such events that change gene expression may be substantial [31]. More generally, lineage-specific sequence is clearly a likely substrate for lineage-specific biology [16], [34], although adaptations to pre-existing functional sequence remain an alternative plausible mode for creating species-specific change [40]. Nevertheless, the sheer ubiquity of sequence turnover, and the clear potential for substantial regulatory change resulting from it, suggests that many aspects of noncoding human biology will not be fully recapitulated by orthologous sequence in eutherian model organisms, including mouse. Thus, our findings could provide a more quantitative basis for assessing the relevance of model organisms to specific questions of human biology.

## Materials and Methods

### Sequence data

We restricted our analyses to genome assemblies that have been sequenced at relatively high coverage, not using for example the 2-fold coverage assemblies of mammalian genomes [41], to minimize the impact of sequencing and assembly errors. From the UCSC Genome Informatics website (http://genome.ucsc.edu/), we acquired softmasked versions of the following genome assemblies: human (hg19), mouse (mm10, mm9, and mm8), rat (rn5), cattle (bosTau7), dog (canFam2), horse (equCab2), guinea pig (cavPor3), rabbit (oryCun2), bushbaby (otoGar3), panda (ailMel1), and rhino (cerSim1). We also acquired a Ferret genome assembly (M_putorius_furo_v1) produced by the Broad Institute. We softmasked the ferret genome assembly using RepeatMasker with carnivore repeat libraries [42].

### Alignment construction and trimming

When available, whole genome pairwise alignments were downloaded from the UCSC Genome Informatics website (http://genome.ucsc.edu). Otherwise, we constructed alignments following UCSC's protocol [43]. Initial alignments were constructed with LASTZ (http://www.bx.psu.edu/miller_lab/), a derivative of BLASTZ [44], and these alignments were subsequently chained and netted using tools from UCSC (Table S1 for alignment parameterisations).

We trimmed each of the whole genome alignments once we found that UCSC alignments contained a minority of poorly aligning sequence (Figure S1, Table S2). Each alignment was rescored to generate a new substitution matrix using a log-odds ratio approach as described previously [45]. We did not impose symmetry on the scoring matrixes with respect to strand or species. We then used the generated substitution matrix, with gap penalties derived from the original alignments, to discard (“trim”) the maximal non-positively scoring terminal segments of the alignment blocks and any non-positively scoring inter-gap segments. Trimming removes terminal and internal alignment segments that are more likely to have arisen under a model of independent evolution than of evolution from a common ancestor. Subsequent analyses were carried out following the discarding of all trimmed sequence. We also excluded alignments that were led by sequence not mapped to chromosomes. We did not exclude non-reciprocally aligning sequence or sequence that lay within known indel hotpot locations as we found removing such sequence had relatively small effects on estimates of α_selIndel_ (Table S3).

### An updated Neutral indel model 1 (NIM1)

The neutral indel model of Lunter *et al.* (2006) [18] (NIM1) estimates the genomic fraction (α_selIndel_) of sequence constrained with respect to indels between a species pair. The model examines the distribution of IGSs from a set of whole genome pairwise alignments using a regression approach over a range of medium IGS lengths to estimate the parameters of a predicted geometric distribution of IGSs in neutral sequence. α_selIndel_ in bp is then estimated by summing up the quantity *x* - 2*K* over all the long IGSs inferred to be in excess of predictions under neutral evolution. Here where *x* is the length of the overrepresented IGS, and *K* is the estimated mean spacing between indels (“neutral overhang”). 20 equally populated G+C content bins are analysed separately to account, in part, for mutational variation that correlates with G+C content. The X chromosome is also analysed separately. A detailed description of the model is given in the original publications [15], [18]. However, two theoretical issues of the model have not been described previously. These are: (A) that thresholding biases the expected lengths of the neutral overhang and, (B) that neutral segments are depleted from the background distribution due to the presence of constrained segments, changing the expected neutral distribution of IGS lengths; resolution of the two issues is described in Text S1.

Our implementation of the NIM1 differs from that of the preceding studies in the manner in which we calculate the bounds of the estimates. The previous approaches constructed the upper and lower bound estimates based on the uncertainty in the degree of clustering of functional elements. The lower bound estimate was derived assuming that functional elements are unclustered (each overrepresented IGS contributes *x* - 2*K* bp towards the α_selIndel_ estimate), while the upper bound was derived assuming a high degree of clustering (each overrepresented IGS contributes *x* - *K* bp). In our revised approach, we construct a 95% confidence interval around the lower *x* - 2*K* bp estimate. The impact of this change on α_selIndel_ estimates can be seen in the simulation study (Table S5). We made this conservative modification to the NIM1 for five reasons: Firstly, the previous upper bound estimate assumes an unrealistically high degree of clustering of functional elements. Secondly, only our modified estimate is always conservative under all the simulation scenarios, whereas the previous implementation of the NIM1 sometimes overestimates the true value of α_selIndel_ (Table S5). Thirdly, altering the clustering of functional elements in the simulations actually has only a minor effect on the estimated quantities of constrained sequence (Figure S11). Fourthly, in addition to the clustering of functional elements, other parameterisations also influenced α_selIndel_ estimates (Table S5), yet the uncertainty in the values of these parameters was not also incorporated into the NIM1 estimate. Instead, we now choose to incorporate the full extent of uncertainty into the simulations. Finally, by providing a 95% confidence interval for the α_selIndel_ estimate of NIM1, we have an estimate that is directly comparable to the NIM2 estimates.

### Estimating the fraction of constraint in subsets of the genome

We have described above how NIM1 is used to estimate the fraction α_selIndel_ of constrained bases within a genome G consisting largely of neutrally evolving sequence. To estimate α_selIndel_ within a subset S⊆G that is not dominated by neutrally evolving sequence, for instance when estimating α_selIndel_ within coding sequence, we instead estimate α_selIndel_ within the subsets G and G\S; the difference between the resulting estimates is the estimate of α_selIndel_ within S.

### Estimating the neutral substitution rates

We extracted ancestral repeat (AR) alignments from the trimmed whole genome alignments using RepeatMasker annotations to identify transposable element and repeat-derived sequence [42]. We then calculated the substitution rate for the alignments using the HKY85 model applied in the PAML package BASEML [46]. We also estimated synonymous substitution rates (dS) across protein coding regions for some species pairs. Estimates of dS for a species pair were made by calculating the median dS of all one-to-one gene orthologs in the Ensembl Compara database with dS<1. Nucleotide substitution rates in AR sequences are very similar to estimates of the synonymous substitution rate (dS) (Figure S5), hence our results appear insensitive to the choice of neutral sequence standard.

### Modelling turnover

The time-homogeneous turnover model makes the following assumptions: for a particular class of functional elements, both the total amount of functional sequence and the rate of turnover are constant in time, and the turnover rate (weighted by the length of the elements) is identical for all elements in the class. Specifically, within a class of functional sites comprising *a* nucleotides, in a small time interval d*t* a number *a b* d*t* of sites dispense with function, while an identical number gain function. Note that to arrive at this result we make an “infinite sites” assumption, namely that the genome can be considered infinitely large compared to *a*; otherwise one would need to account for reversions back to functionality of neutral but previously functional material. Fitting the data to this model under the assumption of independent normally distributed errors in the observations provides estimates and error bounds on parameters *a* and *b*.

### Annotations

Coding sequence for human (hg19), mouse (mm10), and dog (canFam2) and UTR annotations for human (hg19) were obtained from Ensembl version 72 (http://www.ensembl.org/index.html). UTR sequence that overlapped coding sequence was not considered in the UTR analyses. Human (hg19) PhastCons conserved elements were taken from the vertebrate PhastConsElements46way track downloaded from UCSC Genome Informatics (http://genome.ucsc.edu/). Human (hg19) GERP++ conserved elements were downloaded from the Sidow laboratory website (http://mendel.stanford.edu/SidowLab/downloads/gerp/). Repetitive element annotations for all species were taken from RepeatMasker [42]. Other human (hg19) annotations were taken from the ENCODE data available at UCSC Genome Informatics (http://genome.ucsc.edu/ENCODE/). Specifically, the TFBS data and DNase HS data were acquired from the ENCODE clustered merged sets (wgEncodeRegTfbsClusteredV2.bed and wgEncodeRegDnaseClusteredV2.bed respectively). Promoter and enhancer elements were extracted from the ENCODE HMM Chromatin State segmentations tracks, and merged across these samples: wgEncodeBroadHmmGm12878HMM.bed.gz, wgEncodeBroadHmmH1hescHMM.bed.gz, wgEncodeBroadHmmHepg2HMM.bed.gz, wgEncodeBroadHmmHmecHMM.bed.gz, wgEncodeBroadHmmHsmmHMM.bed.gz, wgEncodeBroadHmmHuvecHMM.bed.gz, wgEncodeBroadHmmK562HMM.bed.gz, wgEncodeBroadHmmNhekHMM.bed.gz, and wgEncodeBroadHmmNhlfHMM.bed. We display the results from analysis of the set of Hangauer *et al.* (2013) [21] lncRNAs in Figure 3. We also used the smaller set of ENCODE lncRNAs in Figure S9.

## Supporting Information

Figure S1 Trimming of alignments improves the consistency across alignments. The four different alignments were generated by UCSC with different genome assemblies and under different parameterisations. Of particular significance, the mm8-rn4 and the mm9-rn4(1) alignments used less stringent alignment parameterisations than those used for the mm9-rn4(2) and the mm10-rn5 alignments (Table S1 for all alignment parameterisations). A. α_selIndel_ estimated by the NIM1 on different mouse-rat alignments. The estimates on the alignments trimmed using a log-odds approach (red) are less variable than on the untrimmed alignments (blue). This trend is also observed when α_selIndel_ is estimated with NIM2 (Figure S1). B. The trimmed off sequence is of substantially worse quality then the remaining sequence, as shown by the removed sequence's low sequence identify and high repetitive content. C. Trimming removes more short IGSs from the mm8-rn4/mm9-rn4(1) (mm8-rn4 shown left), than from the mm9-rn4(2)/mm10-rn5 (mm10-rn5, right) alignments.
(TIF)

Figure S2 The quantity of constrained sequence estimated by NIM2 (α_selIndel_) on un-trimmed and trimmed alignments. The trimmed alignments provide more consistent results. This trend is also seen when NIM1 is used to estimate α_selIndel_ (Figure S1A).
(TIF)

Figure S3 The quantity of constrained sequence (α_selIndel_) estimated by NIM1 and NIM2 under different simulation scenarios. NIM1 α_selIndel_ estimates are relatively robust, while NIM2 estimates show a moderate loss of power with increasing divergence.
(TIF)

Figure S4 Quantity of constrained sequence estimated by NIM1 that overlaps sequence identified as conserved by either PhastCons and/or GERP++. Much of the lineage-specific constrained sequence identified by NIM1 is not detected by these other methods that mainly have power to identify pan-mammalian conserved sequences.
(TIF)

Figure S5 Strong positive correlation between ancestral repeat (AR) divergence and synonymous substitution rate (dS). The correlation implies that our results are robust to the choice of neutral standard. The following mammalian species pairs were used: human – cow, human – dog, human – horse, human – mouse, mouse – rat, mouse – cow, mouse – horse, mouse – dog, dog – cow and dog – horse.
(DOCX)

Figure S6 The proportions of coding sequence that are inferred to be under constraint by NIM1 or NIM2 for different pairs of eutherian genomes. NIM1 consistently identifies a greater percentage of coding sequence as being constrained compared to NIM2.
(DOCX)

Figure S7 Sequence constraint over time for different human element types. A. The proportion, and B. the quantity, of annotation bases inferred as being constrained plotted against divergence.
(TIF)

Figure S8 Comparisons of the rates of turnover of different constrained element types. A. P-values are computed by looking at the ratio of observations, which under the hypothesis that the turnover rate is equal, should fit a model with b = 0. B. P-values are computed using a likelihood ratio test to compare a model where the b parameter is shared between the two annotations to one where b is independent for the annotations. C. The same computation as B. except that the length of the NIM1 95% confidence interval were used to calculate the weight for each data point.
(TIF)

Figure S9 The conservation and turnover of ENCODE lncRNAs and a set from Hangauer *et al.* (2013) [21]. A. The proportion of lncRNA bases identified as constrained by NIM1 plotted against the divergence. B. The estimated rates of turnover of the two different lncRNA data sets.
(TIF)

Figure S10 The overlap between different human functional annotations in megabases. The considerable overlap between some annotations has the consequence that evidence of sequence constraint on one type of annotation may instead be attributable to a different annotation that covers the same inter-gap segment.
(TIF)

Figure S11 Quantity of constrained sequence (α_selIndel_) estimated by NIM1 in simulated data under two different scenarios of clustering of functional elements. The estimates were made on simulated sequences of 200 Mb and then scaled (×15) to produce estimates for 3 Gb genomes. The true quantity of constrained sequence is fixed at a scaled value of 150 Mb in every simulation. Varying the clustering coefficient has little effect on estimates of α_selIndel_.
(DOCX)

Table S1 LASTZ parameterisations implemented for the different alignments. BLASTZ parameter names are in parentheses. Rows highlighted in bold represent alignments that we constructed, while the other alignments were constructed by UCSC Genome Informatics.
(DOCX)

Table S2 Sequence quality statistics from different mouse – rat alignments for untrimmed sequence, non-maximally positively scoring sequence trimmed off the starts and ends of alignment blocks, and internally trimmed negatively scoring inter-gap segments. The alignments remaining after trimming are of higher quality than the trimmed-off aligning sequence in the sense that they are both less divergent and consist of proportionally fewer transposable element (TE) derived sequences.
(DOCX)

Table S3 Quantity of constrained sequence (α_selIndel_) estimated by NIM1 on trimmed alignments with alignments processed in one of two ways. Firstly, non-reciprocally aligning sequence was removed, that is sequence that aligns when Species A is the target input and Species B the query input, but not when Species B is the target input and the Species A the query input, or vice-versa. Secondly, indel hotspot regions of the genome were removed. These steps have relatively small effects on estimates of α_selIndel_.
(DOCX)

Table S4 Definitions of parameterisations that were varied across the genome simulations.
(DOCX)

Table S5 The quantity of constrained sequence estimated by NIM1 (α_selIndel_) on simulated data under different paramerisations. The estimates were made on simulated sequences of 200 Mb and then scaled (×15) to produce estimates for genomes of 3 Gb in size. The true quantity of constrained sequence is fixed at a scaled value of 150 Mb in each simulation. Our implementation of NIM1 always estimates α_selIndel_ accurately or conservatively, although there is variation in estimates across the different parameterisations. The previous implementation of the NIM1 by Meader *et al.* (2010) [15] sometimes overestimates α_selIndel_. The parameters for the simulations are provided in Table S4.
(DOCX)

Table S6 The total quantities of constrained sequence estimated in the human genomes at present by different methods. The annotations are mutually exclusive sets as in Figure 4.
(DOCX)

Text S1 A new justification for the Neutral Indel Model 1 (NIM1).
(DOCX)

Text S2 Neutral Indel Model 2 (NIM2).
(DOCX)

Text S3 Alignment trimming improves alignment quality and α_selIndel_ estimates.
(DOCX)

Text S4 Genome simulations demonstrate the accuracy and robustness of the NIMs.
(DOCX)

Text S5 Simulating genome evolution.
(DOCX)

Text S6 Technical artefacts cannot explain observed signatures of turnover.
(DOCX)

Text S7 Modelling turnover of pan-mammalian conserved sequence.
(DOCX)

Text S8 Levels of sequence constraint for protein coding sequences.
(DOCX)
