# Supplementary figures and images for: 8.2% of the Human Genome Is Constrained: Variation in Rates of Turnover across Functional Element Classes in the Human Lineage
Source: PLoS Genet. 2014 Jul 24;10(7):e1004525. doi: 10.1371/journal.pgen.1004525 (PMC4109858; doi:10.1371/journal.pgen.1004525)

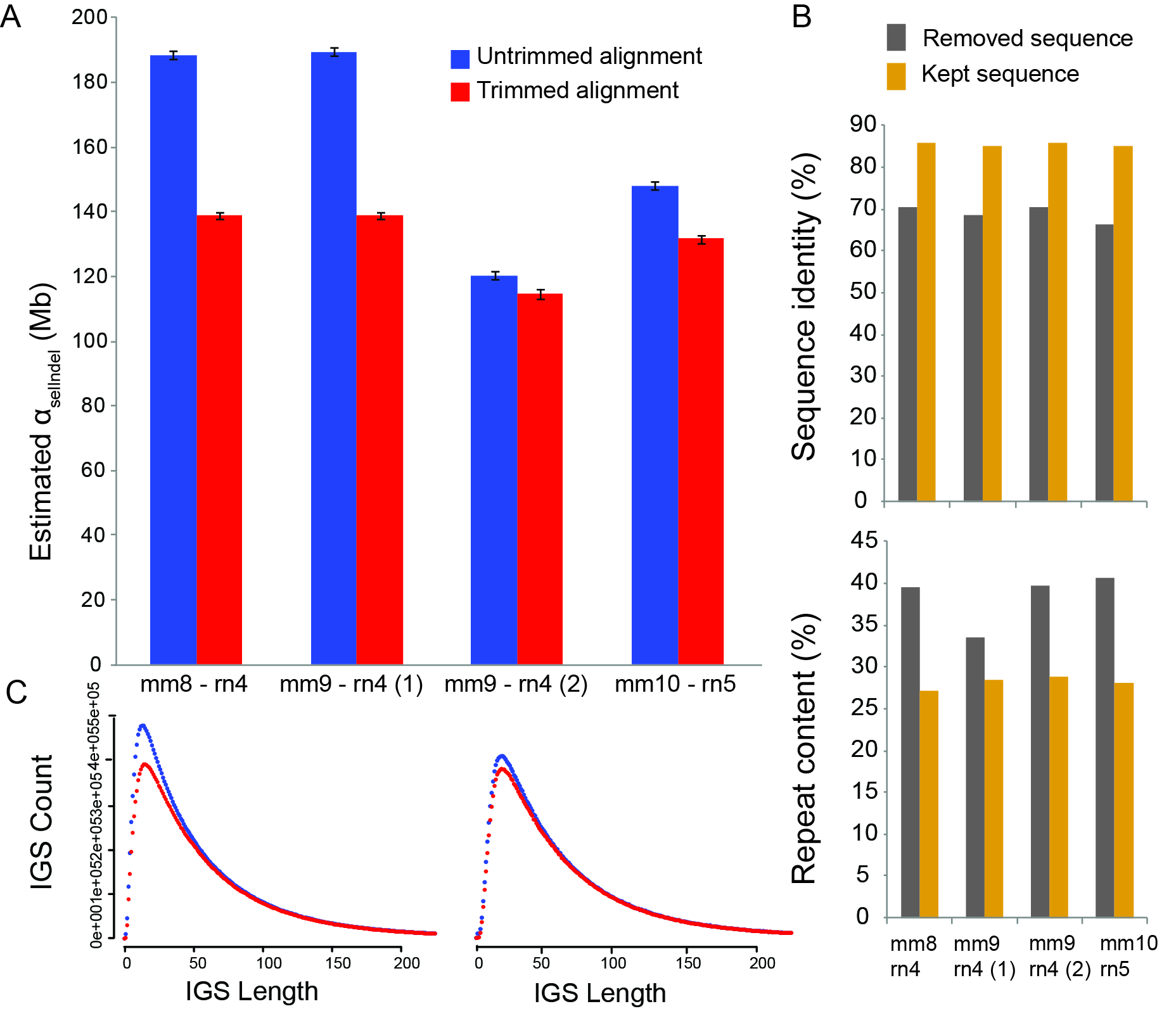

Supplement: Figure S1 — Trimming of alignments improves the consistency across alignments. The four different alignments were generated by UCSC with different genome assemblies and under different parameterisations. Of particular significance, the mm8-rn4 and the mm9-rn4(1) alignments used less stringent alignment parameterisations than those used for the mm9-rn4(2) and the mm10-rn5 alignments (Table S1 for all alignment parameterisations). A. αselIndel estimated by the NIM1 on different mouse-rat alignments. The estimates on the alignments trimmed using a log-odds approach (red) are less variable than on the untrimmed alignments (blue). This trend is also observed when αselIndel is estimated with NIM2 (Figure S1). B. The trimmed off sequence is of substantially worse quality then the remaining sequence, as shown by the removed sequence's low sequence identify and high repetitive content. C. Trimming removes more short IGSs from the mm8-rn4/mm9-rn4(1) (mm8-rn4 shown left), than from the mm9-rn4(2)/mm10-rn5 (mm10-rn5, right) alignments. (TIF) [file pgen.1004525.s001.tif]

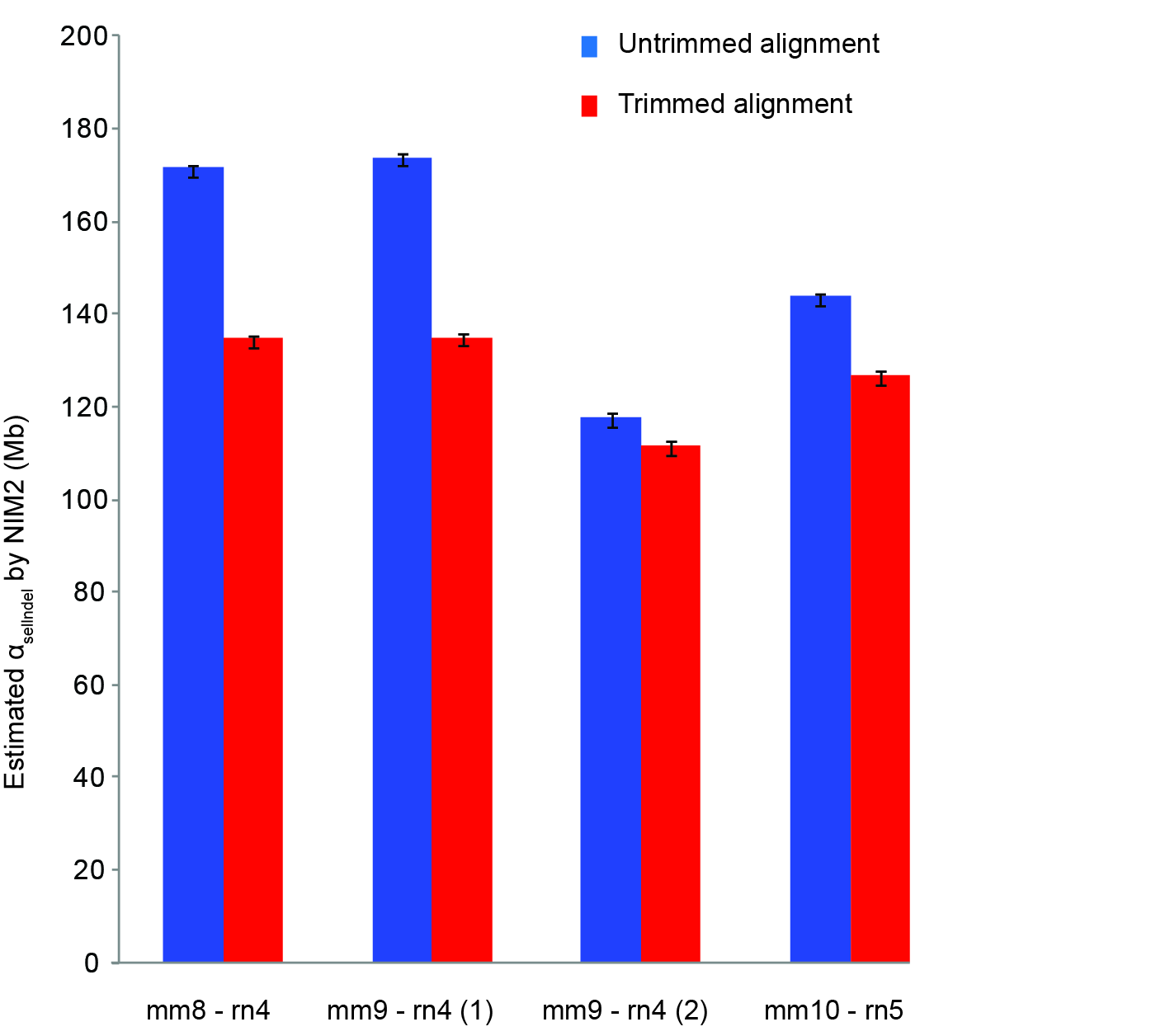

Supplement: Figure S2 — The quantity of constrained sequence estimated by NIM2 (αselIndel) on un-trimmed and trimmed alignments. The trimmed alignments provide more consistent results. This trend is also seen when NIM1 is used to estimate αselIndel (Figure S1A). (TIF) [file pgen.1004525.s002.tif]

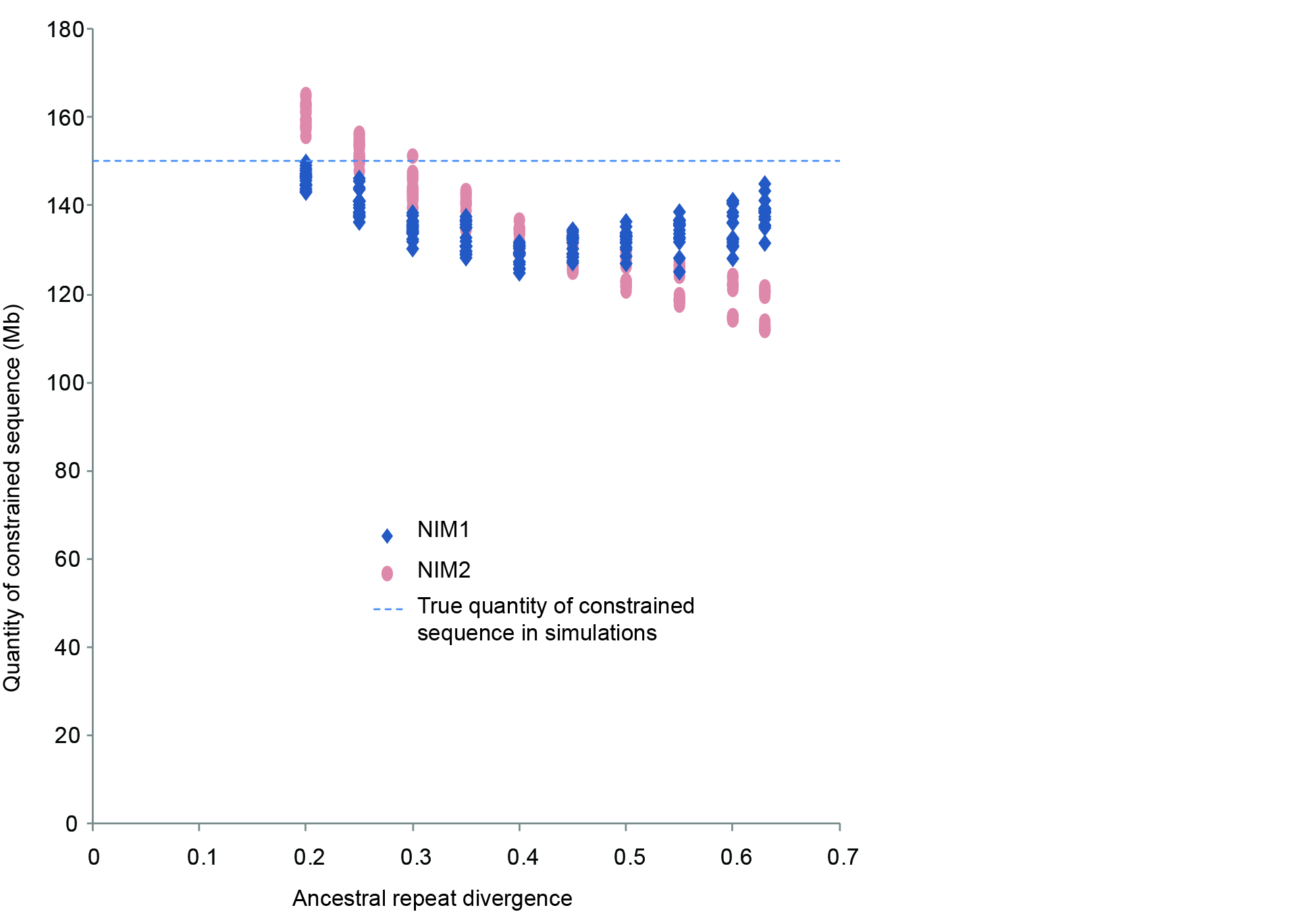

Supplement: Figure S3 — The quantity of constrained sequence (αselIndel) estimated by NIM1 and NIM2 under different simulation scenarios. NIM1 αselIndel estimates are relatively robust, while NIM2 estimates show a moderate loss of power with increasing divergence. (TIF) [file pgen.1004525.s003.tif]

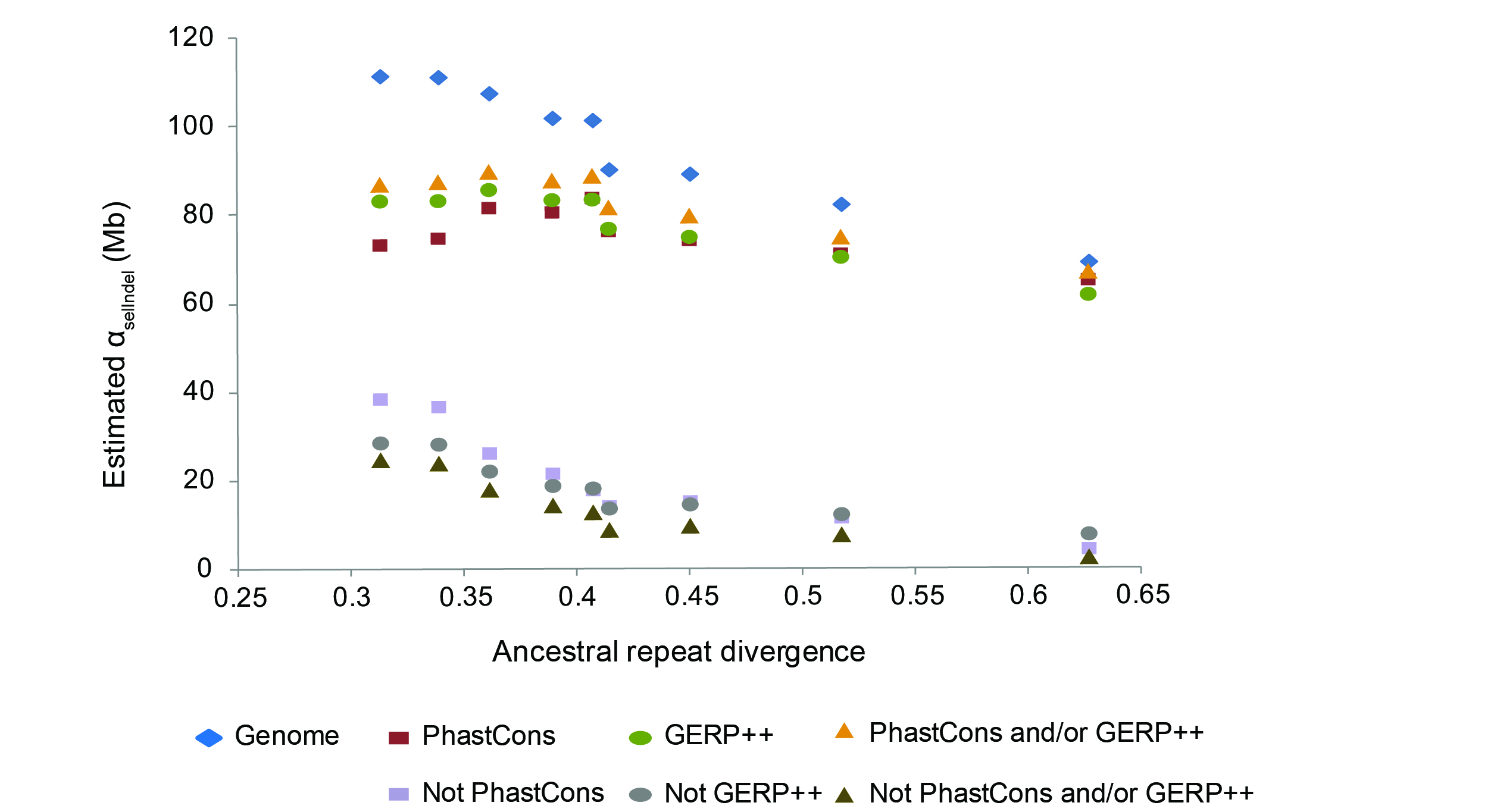

Supplement: Figure S4 — Quantity of constrained sequence estimated by NIM1 that overlaps sequence identified as conserved by either PhastCons and/or GERP++. Much of the lineage-specific constrained sequence identified by NIM1 is not detected by these other methods that mainly have power to identify pan-mammalian conserved sequences. (TIF) [file pgen.1004525.s004.tif]

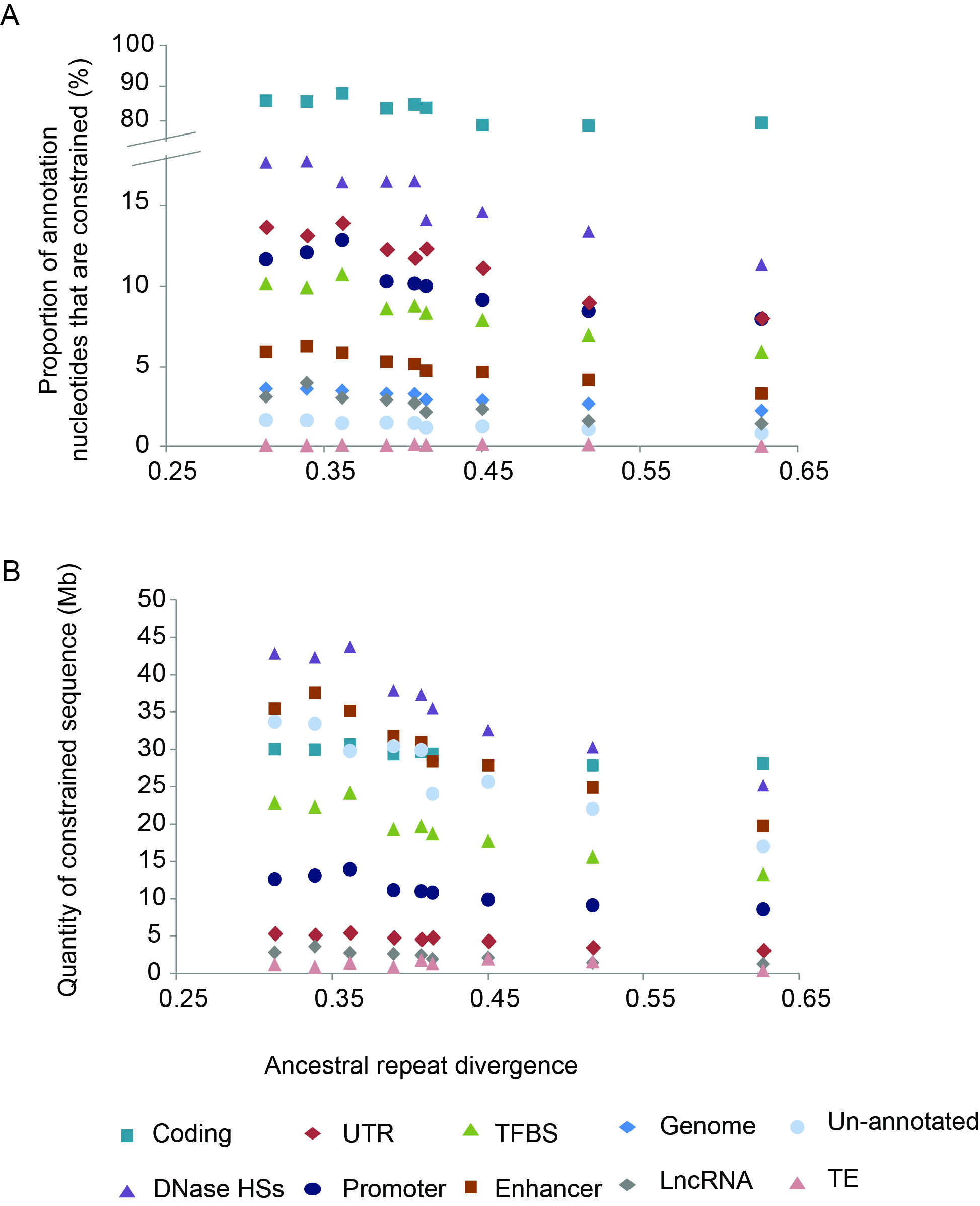

Supplement: Figure S7 — Sequence constraint over time for different human element types. A. The proportion, and B. the quantity, of annotation bases inferred as being constrained plotted against divergence. (TIF) [file pgen.1004525.s007.tif]

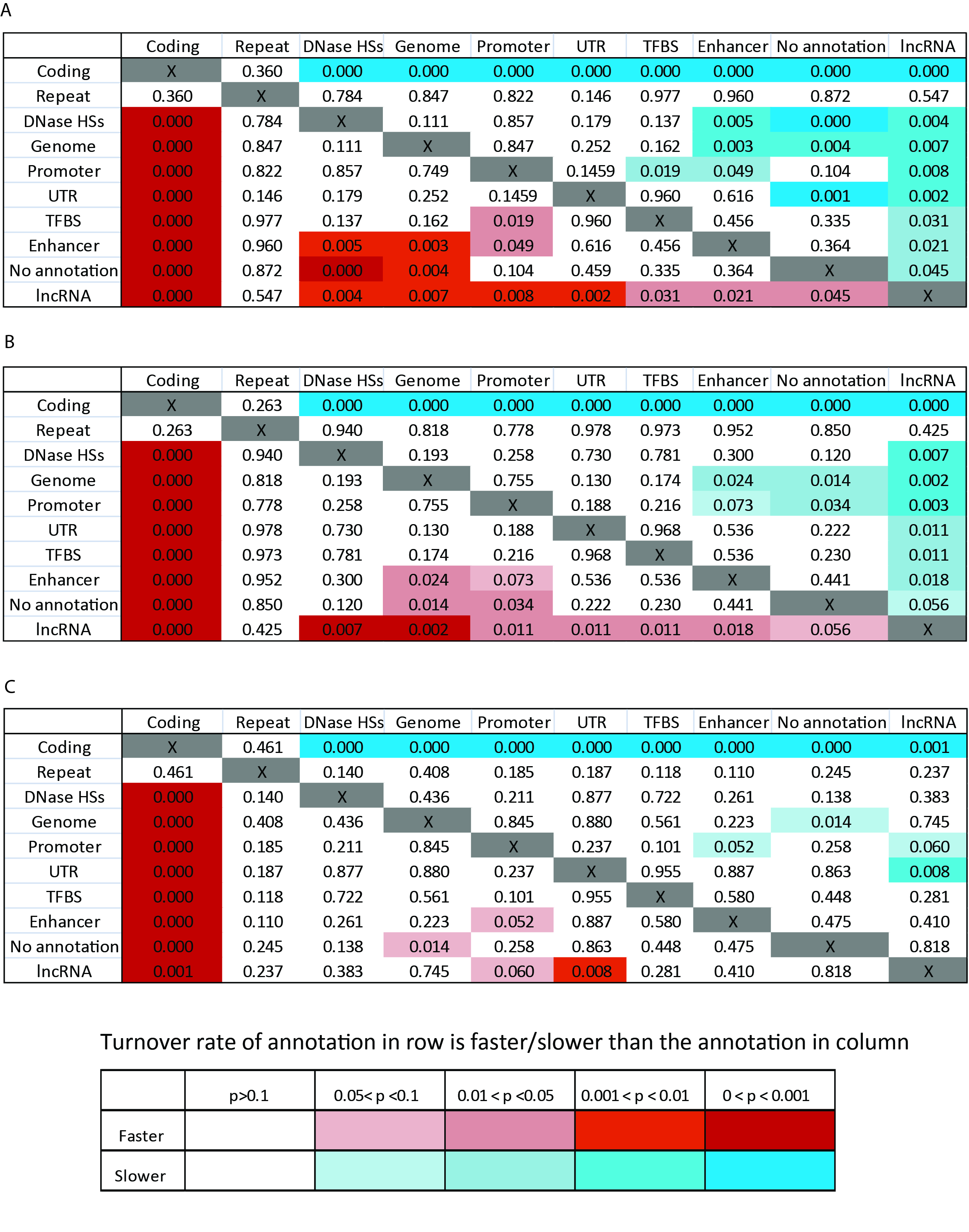

Supplement: Figure S8 — Comparisons of the rates of turnover of different constrained element types. A. P-values are computed by looking at the ratio of observations, which under the hypothesis that the turnover rate is equal, should fit a model with b = 0. B. P-values are computed using a likelihood ratio test to compare a model where the b parameter is shared between the two annotations to one where b is independent for the annotations. C. The same computation as B. except that the length of the NIM1 95% confidence interval were used to calculate the weight for each data point. (TIF) [file pgen.1004525.s008.tif]

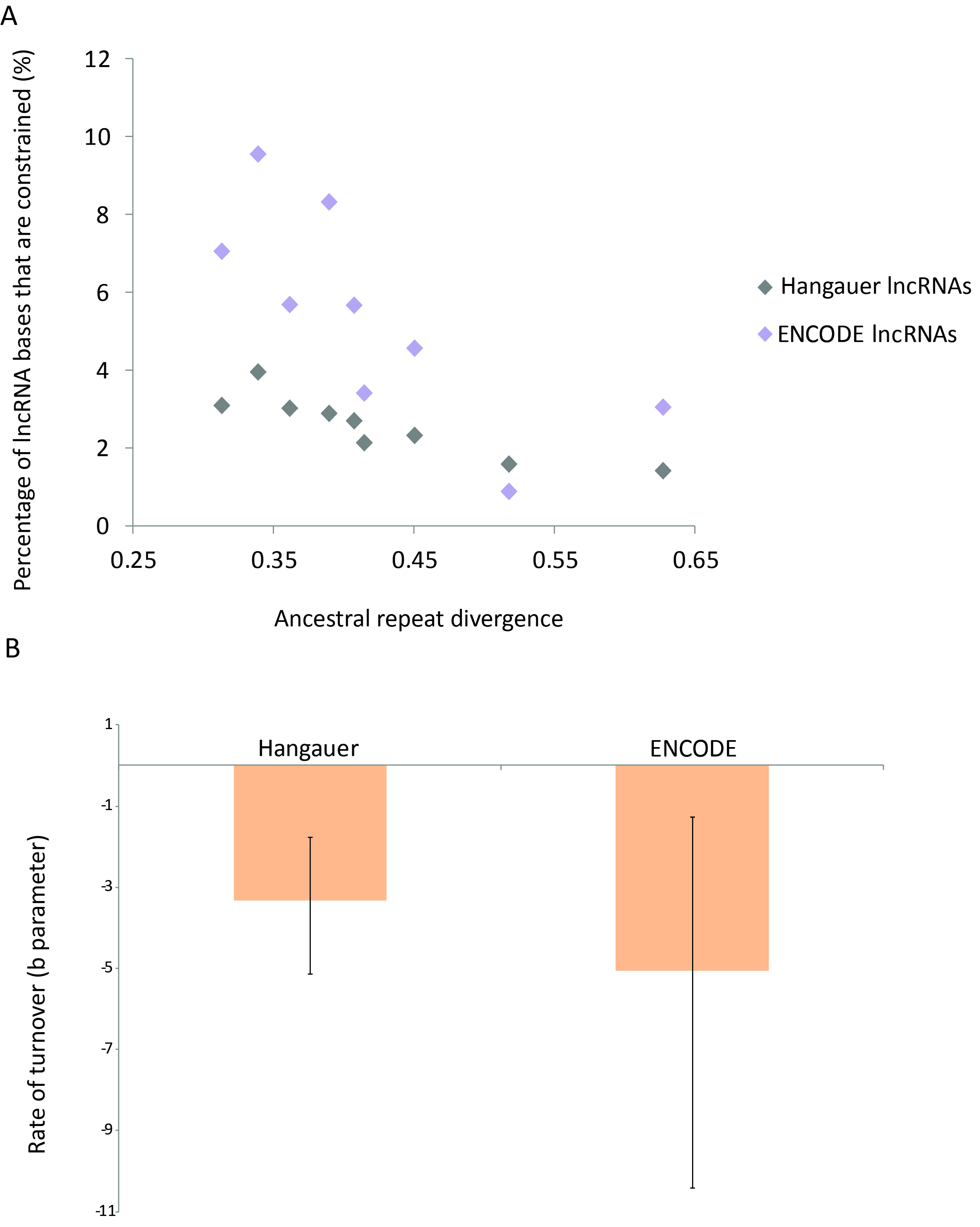

Supplement: Figure S9 — The conservation and turnover of ENCODE lncRNAs and a set from Hangauer et al. (2013) [21]. A. The proportion of lncRNA bases identified as constrained by NIM1 plotted against the divergence. B. The estimated rates of turnover of the two different lncRNA data sets. (TIF) [file pgen.1004525.s009.tif]

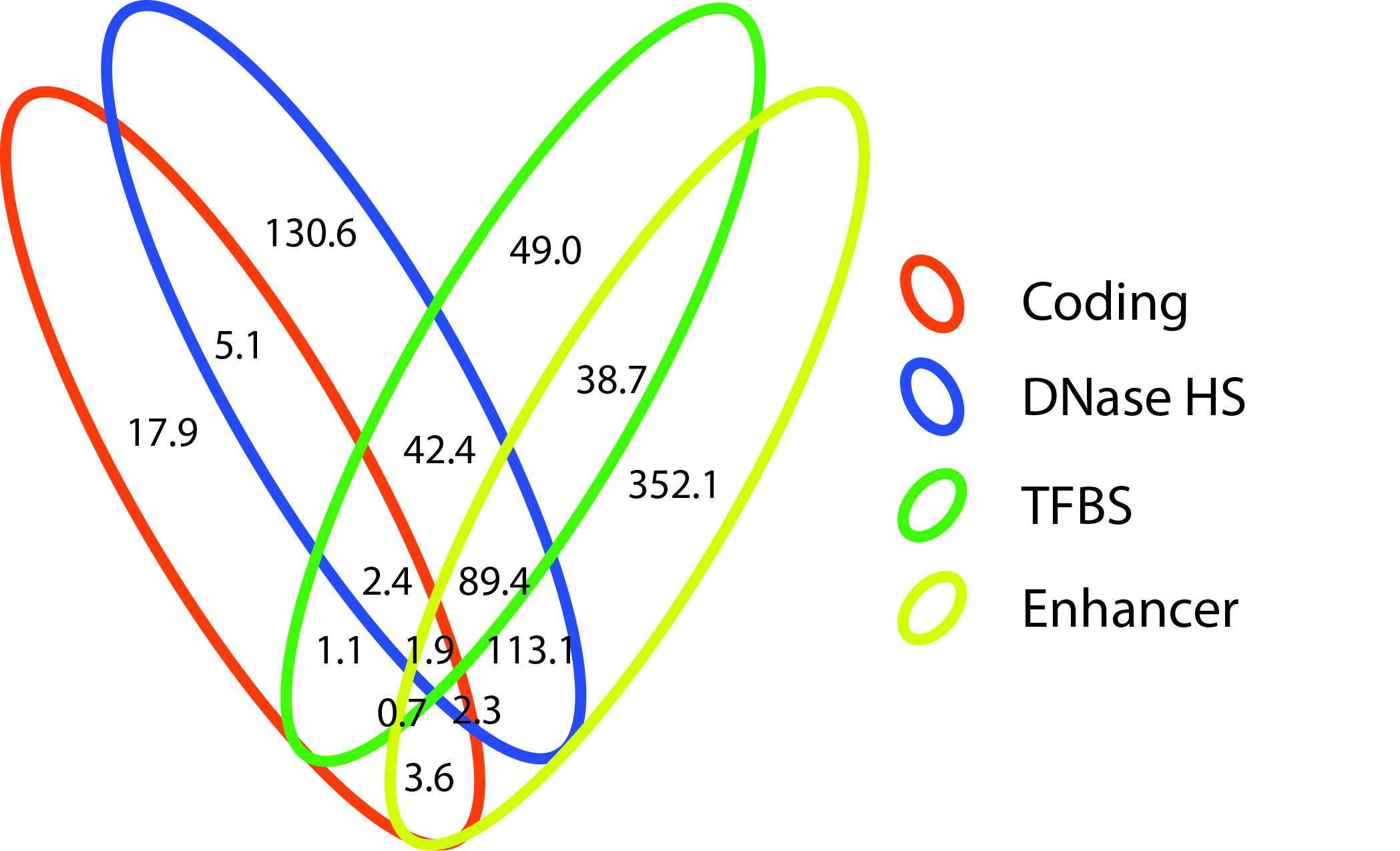

Supplement: Figure S10 — The overlap between different human functional annotations in megabases. The considerable overlap between some annotations has the consequence that evidence of sequence constraint on one type of annotation may instead be attributable to a different annotation that covers the same inter-gap segment. (TIF) [file pgen.1004525.s010.tif]
