# Supplementary material for: 8.2% of the Human Genome Is Constrained: Variation in Rates of Turnover across Functional Element Classes in the Human Lineage
Source: PLoS Genet. 2014 Jul 24;10(7):e1004525. doi: 10.1371/journal.pgen.1004525 (PMC4109858; doi:10.1371/journal.pgen.1004525)
Supplement: Table S1 — LASTZ parameterisations implemented for the different alignments. BLASTZ parameter names are in parentheses. Rows highlighted in bold represent alignments that we constructed, while the other alignments were constructed by UCSC Genome Informatics. (DOCX) [file pgen.1004525.s012.docx]

**Table S1: LASTZ parameterisations implemented for the different alignments. BLASTZ parameter names are in parentheses.** Rows highlighted in bold represent alignments that we constructed, while the other alignments were constructed by UCSC Genome Informatics.

| **Species Pair** | **HSP threshold (K)** | **Gapped threshold (L)** | **Y dropoff (Y)** | **Inner score (H)** | **Gap opening pen (O)** | **Gap extension pen (E)** | **Seed spec (T)** | **Scoring matrix** |
| --- | --- | --- | --- | --- | --- | --- | --- | --- |
| Human – Horse | 3000 | 3000 | 9400 | 0 | 400 | 30 | 1 | Default |
| Human – Rhino | 3000 | 3000 | 9400 | 2000 | 400 | 30 | 1 | Default |
| **Human –Bushbaby** | **3000** | **3000** | **9400** | **2000** | **400** | **30** | **1** | **Default** |
| Human – Dog | 3000 | 3000 | 9400 | 0 | 400 | 30 | 1 | Default |
| Human – Panda | 2200 | 6000 | 3400 | 2000 | 400 | 30 | 2 | Default |
| Human – Cow | 3000 | 3000 | 9400 | 0 | 400 | 30 | 1 | Default |
| Human – Rabbit | 3000 | 3000 | 9400 | 0 | 400 | 30 | 1 | Default |
| Human – Guinea pig | 3000 | 2200 | 940 | 2000 | 400 | 30 | 1 | Default |
| Human – Mouse | 3000 | 2200 | 9400 | 2000 | 400 | 30 | 1 | Default |
| Mouse – Rat  (mm8-rn4) | 3000 | 2200 | 9400 | 2000 | 400 | 30 | 1 | Default |
| Mouse – Rat  (mm9-rn4 (1)) | 3000 | 2200 | 9400 | 2000 | 400 | 30 | 1 | Default |
| Mouse – Rat  (mm9-rn4 (2)) | 4500 | 2200 | 15000 | 2000 | 600 | 55 | 2 | MR_old |
| Mouse – Rat  (mm10-rn5) | 3000 | 3000 | 5000 | 2000 | 600 | 55 | 2 | MR_op |
| **Mouse – Horse** | **3000** | **3000** | **9400** | **2000** | **400** | **30** | **1** | **Default** |
| **Mouse – Dog** | **3000** | **3000** | **9400** | **2000** | **400** | **30** | **1** | **Default** |
| Mouse – Cow | 3000 | 3000 | 9400 | 2000 | 400 | 30 | 1 | Default |
| **Dog** – **Ferret** | **3000** | **3000** | **5000** | **2000** | **600** | **55** | **2** | **MR_op** |
| Dog – Horse | 3000 | 2200 | 9400 | 2000 | 400 | 20 | 1 | Default |
| Dog –  Cow | 3000 | 2200 | 9400 | 2000 | 400 | 30 | 1 | Default |

Default scoring matrix

A     C   G     T
A  91  -114  -31  -123
C -114  100 -125   -31
G  -31 -125  100  -114
T -123  -31 -114    91

MR_old scoring matrix

A     C   G     T

A 56 -109 -45 -137

C -109 100 -103 -45

G -45 -103 100 -109

T -137 -45 -109 56

MR_op scoring matrix

A     C   G     T

A 100 -139 -54 -170

C -139 95 -83 -54

G -54 -83 95 -139

T -170 -54 -139 100
