# Supplementary material for: 8.2% of the Human Genome Is Constrained: Variation in Rates of Turnover across Functional Element Classes in the Human Lineage
Source: PLoS Genet. 2014 Jul 24;10(7):e1004525. doi: 10.1371/journal.pgen.1004525 (PMC4109858; doi:10.1371/journal.pgen.1004525)
Supplement: Table S2 — Sequence quality statistics from different mouse – rat alignments for untrimmed sequence, non-maximally positively scoring sequence trimmed off the starts and ends of alignment blocks, and internally trimmed negatively scoring inter-gap segments. The alignments remaining after trimming are of higher quality than the trimmed-off aligning sequence in the sense that they are both less divergent and consist of proportionally fewer transposable element (TE) derived sequences. (DOCX) [file pgen.1004525.s013.docx]

**Table S2: Sequence quality statistics from different mouse – rat alignments for untrimmed sequence, non-maximally positively scoring sequence trimmed off the starts and ends of alignment blocks, and internally trimmed negatively scoring inter-gap segments.** The alignments remaining after trimming are of higher quality than the trimmed-off aligning sequence in the sense that they are both less divergent and consist of proportionally fewer transposable element (TE) derived sequences.

| **Sequence type** | **Ungapped sequence length (Mb)** | | | | **Substitution Divergence** | | | |
| --- | --- | --- | --- | --- | --- | --- | --- | --- |
|  | **mm8**  **rn4** | **mm9**  **rn4 (1)** | **mm9**  **rn4 (2)** | **mm10**  **rn5** | **mm8**  **rn4** | **mm9**  **rn4 (1)** | **mm9**  **rn4 (2)** | **mm10**  **rn5** |
| **Remaining**  **sequence** | 1681 | 1696 | 1689 | 1743 | 0.144 | 0.145 | 0.150 | 0.148 |
| **Trimmed start** | 46.00 | 48.42 | 8.441 | 17.88 | 0.280 | 0.281 | 0.309 | 0.295 |
| **Trimmed end** | 46.61 | 49.08 | 8.695 | 18.46 | 0.280 | 0.281 | 0.312 | 0.295 |
| **Trimmed internal** | 7.649 | 7.857 | 3.415 | 4.673 | 0.473 | 0.473 | 0.463 | 0.465 |

| **Sequence type** | **Mean IGS length (bp)** | | | | **Proportion TE** | | | |
| --- | --- | --- | --- | --- | --- | --- | --- | --- |
|  | **mm8**  **rn4** | **mm9**  **rn4 (1)** | **mm9**  **rn4 (2)** | **mm10**  **rn5** | **mm8**  **rn4** | **mm9**  **rn4 (1)** | **mm9**  **rn4 (2)** | **mm10**  **rn5** |
| **Remaining**  **sequence** | 64.2 | 64.3 | 71.8 | 68.3 | 0.272 | 0.287 | 0.281 | 0.284 |
| **Trimmed start** | 22.4 | 22.6 | 20.6 | 20.8 | 0.395 | 0.396 | 0.411 | 0.329 |
| **Trimmed end** | 22.3 | 22.4 | 20.0 | 20.0 | 0.394 | 0.395 | 0.408 | 0.333 |
| **Trimmed internal** | 25.3 | 25.5 | 32.7 | 26.9 | 0.416 | 0.412 | 0.387 | 0.372 |
