# Supplementary material for: 8.2% of the Human Genome Is Constrained: Variation in Rates of Turnover across Functional Element Classes in the Human Lineage
Source: PLoS Genet. 2014 Jul 24;10(7):e1004525. doi: 10.1371/journal.pgen.1004525 (PMC4109858; doi:10.1371/journal.pgen.1004525)
Supplement: Table S3 — Quantity of constrained sequence (αselIndel) estimated by NIM1 on trimmed alignments with alignments processed in one of two ways. Firstly, non-reciprocally aligning sequence was removed, that is sequence that aligns when Species A is the target input and Species B the query input, but not when Species B is the target input and the Species A the query input, or vice-versa. Secondly, indel hotspot regions of the genome were removed. These steps have relatively small effects on estimates of αselIndel. (DOCX) [file pgen.1004525.s014.docx]

**Table S3: Quantity of constrained sequence (α_selIndel_) estimated by NIM1 on trimmed alignments with alignments processed in one of two ways.** Firstly, non-reciprocally aligning sequence was removed, that is sequence that aligns when Species A is the target input and Species B the query input, but not when Species B is the target input and the Species A the query input, or vice-versa. Secondly, indel hotspot regions of the genome were removed. These steps have relatively small effects on estimates of α_selIndel_.

| **Condition** | **Species Pair** | **Estimate of α_selIndel_** | **Aligning sequence (Mb)** |
| --- | --- | --- | --- |
| Only reciprocally aligning sequence | Mouse – Rat | 136.0 | 1607.4 |
|  | Human – Mouse | 62.6 | 751.7 |
| No indel hotpot regions | Mouse – Rat | 138.3 | 1707.7 |
|  | Human – Horse | 110.7 | 1545.8 |
|  | Human – Rhino | 111.7 | 1578.2 |
|  | Human – Bushbaby | 108.3 | 1461.7 |
|  | Human – Dog | 101.4 | 1416.8 |
|  | Human – Panda | 102.6 | 1390.7 |
|  | Human – Cow | 90.5 | 1239.1 |
|  | Human – Rabbit | 89.2 | 1160.9 |
|  | Human – Guinea pig | 82.9 | 1132.9 |
|  | Human – Mouse | 69.4 | 864.2 |
