# Supplementary material for: 8.2% of the Human Genome Is Constrained: Variation in Rates of Turnover across Functional Element Classes in the Human Lineage
Source: PLoS Genet. 2014 Jul 24;10(7):e1004525. doi: 10.1371/journal.pgen.1004525 (PMC4109858; doi:10.1371/journal.pgen.1004525)
Supplement: Table S4 — Definitions of parameterisations that were varied across the genome simulations. (DOCX) [file pgen.1004525.s015.docx]

**Table S4: Definitions of parameterisations that were varied across the genome simulations.**

| **Parameter Name** | **Parameter Description** |
| --- | --- |
| Species divergence | Mean proportion of substitutions per site that have changed between the species pair |
| Functional element clustering coefficient | Proportion of functional segments that are followed by another |
| Indel fixation probability | Fixation probability of an indel touching a functional segment |
| Functional expected length | Expected length of functional segment drawn from the gamma distribution |
| Functional shape | Shape parameter of gamma distribution for functional material |
| Intervening expected length | Expected length of neutral intervening segment drawn from the gamma distribution |
| Intervening shape | Shape parameter of gamma distribution for intervening neutral material |
| \| Substitution/indel ratio \|  \| \| --- \| --- \| | The number of times higher the substitution rate was compared to the indel rate |
| Residual indel rate variation | This parameter is a value from 0 to 1 which allows the neutral indel rate to vary per simulated block within a set of boundaries. For example, if you set it to 0.5, for a neutral indel rate of 0.2, there would be a boundary around that 0.2 (in this case 0.1-0.3) from which the neutral indel rate is picked for each simulated block. |
