# Supplementary material for: 8.2% of the Human Genome Is Constrained: Variation in Rates of Turnover across Functional Element Classes in the Human Lineage
Source: PLoS Genet. 2014 Jul 24;10(7):e1004525. doi: 10.1371/journal.pgen.1004525 (PMC4109858; doi:10.1371/journal.pgen.1004525)
Supplement: Table S5 — The quantity of constrained sequence estimated by NIM1 (αselIndel) on simulated data under different paramerisations. The estimates were made on simulated sequences of 200 Mb and then scaled (×15) to produce estimates for genomes of 3 Gb in size. The true quantity of constrained sequence is fixed at a scaled value of 150 Mb in each simulation. Our implementation of NIM1 always estimates αselIndel accurately or conservatively, although there is variation in estimates across the different parameterisations. The previous implementation of the NIM1 by Meader et al. (2010) [15] sometimes overestimates αselIndel. The parameters for the simulations are provided in Table S4. (DOCX) [file pgen.1004525.s016.docx]

**Table S5: The quantity of constrained sequence estimated by NIM1 (α_selIndel_) on simulated data under different paramerisations.** The estimates were made on simulated sequences of 200Mb and then scaled (x15) to produce estimates for genomes of 3Gb in size. The true quantity of constrained sequence is fixed at a scaled value of 150Mb in each simulation. Our implementation of NIM1 always estimates α_selIndel_ accurately or conservatively, although there is variation in estimates across the different parameterisations. The previous implementation of the NIM1 by Meader *et al.* (2010) [[2](#_ENREF_2)] sometimes overestimates α_selIndel ._ The parameters for the simulations are provided in Table S4.

| **NIM1 estimate of α_selIndel_ (Mb)** | **Upper bound estimate of α_selIndel_ (Mb) under Meader et al. (2010) implementation** | **Parameterisation** | | | | |
| --- | --- | --- | --- | --- | --- | --- |
|  |  | **Divergence** | **Clustering**  **Coefficient** | **Functional**  **Shape** | **Intervening**  **Expected**  **Length (bp)** | **Intervening**  **Shape** |
| 133.9 | 160.9 | 0.5 | 0.5 | 2 | 25 | 2 |
| 138.9 | 164.3 | 0.63 | 0.5 | 2 | 15 | 1 |
| 135.3 | 161.3 | 0.5 | 0.5 | 2 | 15 | 2 |
| 134.5 | 159.3 | 0.45 | 0.5 | 1 | 15 | 2 |
| 133.4 | 160.3 | 0.45 | 0.5 | 2 | 25 | 2 |
| 132.8 | 158.1 | 0.45 | 0.5 | 1 | 25 | 1 |
| 135.9 | 161.6 | 0.55 | 0.5 | 1 | 25 | 2 |
| 136.5 | 162.1 | 0.55 | 0.5 | 2 | 15 | 1 |
| 135.6 | 161.0 | 0.55 | 0.5 | 2 | 15 | 2 |
| 131.8 | 155.6 | 0.55 | 0.5 | 2 | 25 | 1 |
| 128.1 | 151.1 | 0.55 | 0.5 | 2 | 25 | 2 |
| 133.3 | 157.2 | 0.5 | 0.5 | 1 | 15 | 1 |
| 132.7 | 155.7 | 0.6 | 0.5 | 1 | 15 | 2 |
| 131.7 | 154.3 | 0.6 | 0.5 | 1 | 25 | 1 |
| 132.4 | 155.2 | 0.6 | 0.5 | 1 | 25 | 2 |
| 136.1 | 160.8 | 0.6 | 0.5 | 2 | 15 | 1 |
| 135.4 | 160.2 | 0.63 | 0.5 | 2 | 25 | 2 |
| 128.6 | 151.3 | 0.5 | 0.5 | 1 | 25 | 2 |
| 130.1 | 152.8 | 0.5 | 0.5 | 2 | 15 | 1 |
| 134.3 | 165.0 | 0.3 | 0.5 | 2 | 25 | 2 |
| 131.9 | 157.2 | 0.35 | 0.5 | 1 | 15 | 1 |
| 128.2 | 150.9 | 0.35 | 0.5 | 1 | 15 | 2 |
| 132.8 | 159.1 | 0.35 | 0.5 | 1 | 25 | 1 |
| 133.9 | 162.5 | 0.3 | 0.5 | 1 | 25 | 2 |
| 137.8 | 168.8 | 0.3 | 0.5 | 2 | 15 | 1 |
| 130.3 | 157.5 | 0.3 | 0.5 | 2 | 15 | 2 |
| 136.6 | 167.9 | 0.3 | 0.5 | 2 | 25 | 1 |
| 132.6 | 157.8 | 0.45 | 0.5 | 2 | 15 | 2 |
| 134.1 | 161.2 | 0.45 | 0.5 | 2 | 25 | 1 |
| 128.3 | 150.8 | 0.45 | 0.5 | 1 | 25 | 2 |
| 132.9 | 158.0 | 0.45 | 0.5 | 2 | 15 | 1 |
| 136.8 | 165.1 | 0.35 | 0.5 | 1 | 25 | 2 |
| 129.7 | 154.5 | 0.35 | 0.5 | 2 | 15 | 1 |
| 131.7 | 158.1 | 0.4 | 0.5 | 2 | 25 | 2 |
| 131.6 | 155.6 | 0.45 | 0.5 | 1 | 15 | 1 |
| 144.6 | 183.8 | 0.2 | 0.5 | 1 | 15 | 1 |
| 144.9 | 183.1 | 0.2 | 0.5 | 1 | 25 | 1 |
| 148.0 | 187.9 | 0.2 | 0.5 | 1 | 15 | 2 |
| 144.6 | 185.2 | 0.2 | 0.5 | 2 | 15 | 1 |
| 143.9 | 184.2 | 0.2 | 0.5 | 1 | 25 | 2 |
| 146.7 | 191.0 | 0.2 | 0.5 | 2 | 25 | 1 |
| 149.9 | 195.3 | 0.2 | 0.5 | 2 | 15 | 2 |
| 141.0 | 172.7 | 0.25 | 0.5 | 1 | 15 | 1 |
| 145.7 | 187.4 | 0.2 | 0.5 | 2 | 25 | 2 |
| 137.5 | 163.0 | 0.63 | 0.5 | 2 | 15 | 2 |
| 132.9 | 157.1 | 0.5 | 0.5 | 1 | 25 | 1 |
| 129.3 | 154.0 | 0.4 | 0.5 | 2 | 25 | 1 |
| 131.1 | 152.2 | 0.6 | 0.5 | 1 | 15 | 1 |
| 139.4 | 165.6 | 0.63 | 0.5 | 1 | 25 | 2 |
| 143.4 | 171.3 | 0.63 | 0.5 | 1 | 25 | 1 |
| 127.3 | 150.2 | 0.4 | 0.5 | 2 | 15 | 2 |
| 138.7 | 163.5 | 0.63 | 0.5 | 1 | 15 | 1 |
| 137.7 | 164.3 | 0.6 | 0.5 | 2 | 25 | 2 |
| 130.7 | 154.2 | 0.6 | 0.5 | 2 | 25 | 1 |
| 132.3 | 155.7 | 0.6 | 0.5 | 2 | 15 | 2 |
| 135.5 | 160.3 | 0.55 | 0.5 | 1 | 25 | 1 |
| 138.5 | 164.7 | 0.63 | 0.5 | 2 | 25 | 1 |
| 136.7 | 161.5 | 0.55 | 0.5 | 1 | 15 | 2 |
| 139.5 | 173.1 | 0.25 | 0.5 | 1 | 25 | 1 |
| 143.7 | 178.3 | 0.25 | 0.5 | 1 | 15 | 2 |
| 145.6 | 182.8 | 0.25 | 0.5 | 2 | 15 | 1 |
| 137.6 | 171.1 | 0.25 | 0.5 | 1 | 25 | 2 |
| 138.4 | 173.0 | 0.25 | 0.5 | 2 | 25 | 1 |
| 146.2 | 186.1 | 0.25 | 0.5 | 2 | 15 | 2 |
| 136.3 | 163.7 | 0.3 | 0.5 | 1 | 15 | 1 |
| 137.9 | 173.5 | 0.25 | 0.5 | 2 | 25 | 2 |
| 135.7 | 165.2 | 0.3 | 0.5 | 1 | 25 | 1 |
| 134.4 | 161.8 | 0.3 | 0.5 | 1 | 15 | 2 |
| 137.6 | 168.0 | 0.35 | 0.5 | 2 | 25 | 1 |
| 135.0 | 162.9 | 0.35 | 0.5 | 2 | 15 | 2 |
| 126.7 | 149.1 | 0.4 | 0.5 | 2 | 15 | 1 |
| 130.8 | 156.0 | 0.4 | 0.5 | 1 | 25 | 2 |
| 125.8 | 148.0 | 0.4 | 0.5 | 1 | 25 | 1 |
| 128.8 | 151.7 | 0.4 | 0.5 | 1 | 15 | 2 |
| 129.1 | 152.0 | 0.4 | 0.5 | 1 | 15 | 1 |
| 126.9 | 149.0 | 0.5 | 0.5 | 2 | 25 | 1 |
| 141.2 | 167.2 | 0.63 | 0.5 | 1 | 15 | 2 |
| 135.1 | 165.3 | 0.35 | 0.5 | 2 | 25 | 2 |
| 135.9 | 160.8 | 0.55 | 0.5 | 1 | 15 | 1 |
| 133.0 | 156.7 | 0.5 | 0.5 | 1 | 15 | 2 |
| 133.1 | 161.1 | 0.5 | 0.1 | 2 | 25 | 2 |
| 135.0 | 160.6 | 0.63 | 0.1 | 2 | 15 | 1 |
| 133.8 | 161.2 | 0.5 | 0.1 | 2 | 15 | 2 |
| 132.3 | 159.7 | 0.45 | 0.1 | 1 | 15 | 2 |
| 129.2 | 156.5 | 0.45 | 0.1 | 2 | 25 | 2 |
| 127.5 | 152.1 | 0.45 | 0.1 | 1 | 25 | 1 |
| 133.7 | 159.8 | 0.55 | 0.1 | 1 | 25 | 2 |
| 134.5 | 161.3 | 0.55 | 0.1 | 2 | 15 | 1 |
| 135.9 | 163.8 | 0.55 | 0.1 | 2 | 15 | 2 |
| 132.5 | 158.6 | 0.55 | 0.1 | 2 | 25 | 1 |
| 136.8 | 165.5 | 0.55 | 0.1 | 2 | 25 | 2 |
| 130.6 | 155.6 | 0.5 | 0.1 | 1 | 15 | 1 |
| 128.1 | 150.1 | 0.6 | 0.1 | 1 | 15 | 2 |
| 141.0 | 170.4 | 0.6 | 0.1 | 1 | 25 | 1 |
| 140.4 | 169.6 | 0.6 | 0.1 | 1 | 25 | 2 |
| 140.6 | 169.9 | 0.6 | 0.1 | 2 | 15 | 1 |
| 145.0 | 176.7 | 0.63 | 0.1 | 2 | 25 | 2 |
| 131.8 | 157.7 | 0.5 | 0.1 | 1 | 25 | 2 |
| 131.5 | 158.8 | 0.5 | 0.1 | 2 | 15 | 1 |
| 133.7 | 167.2 | 0.3 | 0.1 | 2 | 25 | 2 |
| 129.7 | 156.9 | 0.35 | 0.1 | 1 | 15 | 1 |
| 130.9 | 158.8 | 0.35 | 0.1 | 1 | 15 | 2 |
| 129.1 | 156.2 | 0.35 | 0.1 | 1 | 25 | 1 |
| 134.8 | 167.3 | 0.3 | 0.1 | 1 | 25 | 2 |
| 135.3 | 169.8 | 0.3 | 0.1 | 2 | 15 | 1 |
| 133.8 | 167.5 | 0.3 | 0.1 | 2 | 15 | 2 |
| 138.4 | 176.0 | 0.3 | 0.1 | 2 | 25 | 1 |
| 130.2 | 157.8 | 0.45 | 0.1 | 2 | 15 | 2 |
| 127.1 | 152.7 | 0.45 | 0.1 | 2 | 25 | 1 |
| 129.0 | 154.1 | 0.45 | 0.1 | 1 | 25 | 2 |
| 128.4 | 155.0 | 0.45 | 0.1 | 2 | 15 | 1 |
| 130.7 | 159.3 | 0.35 | 0.1 | 1 | 25 | 2 |
| 136.5 | 170.0 | 0.35 | 0.1 | 2 | 15 | 1 |
| 131.3 | 160.6 | 0.4 | 0.1 | 2 | 25 | 2 |
| 132.7 | 160.2 | 0.45 | 0.1 | 1 | 15 | 1 |
| 149.1 | 197.1 | 0.2 | 0.1 | 1 | 15 | 1 |
| 147.0 | 193.8 | 0.2 | 0.1 | 1 | 25 | 1 |
| 147.4 | 194.4 | 0.2 | 0.1 | 1 | 15 | 2 |
| 143.1 | 190.5 | 0.2 | 0.1 | 2 | 15 | 1 |
| 146.6 | 192.2 | 0.2 | 0.1 | 1 | 25 | 2 |
| 143.4 | 191.2 | 0.2 | 0.1 | 2 | 25 | 1 |
| 146.2 | 194.5 | 0.2 | 0.1 | 2 | 15 | 2 |
| 138.6 | 175.5 | 0.25 | 0.1 | 1 | 15 | 1 |
| 148.5 | 201.8 | 0.2 | 0.1 | 2 | 25 | 2 |
| 131.5 | 155.7 | 0.63 | 0.1 | 2 | 15 | 2 |
| 132.3 | 158.9 | 0.5 | 0.1 | 1 | 25 | 1 |
| 127.3 | 153.9 | 0.4 | 0.1 | 2 | 25 | 1 |
| 141.2 | 170.3 | 0.6 | 0.1 | 1 | 15 | 1 |
| 139.4 | 166.8 | 0.63 | 0.1 | 1 | 25 | 2 |
| 136.9 | 162.9 | 0.63 | 0.1 | 1 | 25 | 1 |
| 129.2 | 156.3 | 0.4 | 0.1 | 2 | 15 | 2 |
| 138.4 | 164.9 | 0.63 | 0.1 | 1 | 15 | 1 |
| 138.5 | 167.3 | 0.6 | 0.1 | 2 | 25 | 2 |
| 130.8 | 155.1 | 0.6 | 0.1 | 2 | 25 | 1 |
| 136.2 | 163.3 | 0.6 | 0.1 | 2 | 15 | 2 |
| 138.6 | 167.2 | 0.55 | 0.1 | 1 | 25 | 1 |
| 135.6 | 162.4 | 0.63 | 0.1 | 2 | 25 | 1 |
| 125.0 | 146.3 | 0.55 | 0.1 | 1 | 15 | 2 |
| 141.1 | 179.8 | 0.25 | 0.1 | 1 | 25 | 1 |
| 141.0 | 179.5 | 0.25 | 0.1 | 1 | 15 | 2 |
| 140.2 | 179.4 | 0.25 | 0.1 | 2 | 15 | 1 |
| 136.3 | 172.3 | 0.25 | 0.1 | 1 | 25 | 2 |
| 144.1 | 186.8 | 0.25 | 0.1 | 2 | 25 | 1 |
| 141.0 | 182.0 | 0.25 | 0.1 | 2 | 15 | 2 |
| 135.3 | 167.7 | 0.3 | 0.1 | 1 | 15 | 1 |
| 137.4 | 176.3 | 0.25 | 0.1 | 2 | 25 | 2 |
| 131.9 | 162.5 | 0.3 | 0.1 | 1 | 25 | 1 |
| 132.4 | 163.1 | 0.3 | 0.1 | 1 | 15 | 2 |
| 135.8 | 169.4 | 0.35 | 0.1 | 2 | 25 | 1 |
| 128.9 | 157.9 | 0.35 | 0.1 | 2 | 15 | 2 |
| 130.8 | 159.1 | 0.4 | 0.1 | 2 | 15 | 1 |
| 124.8 | 149.2 | 0.4 | 0.1 | 1 | 25 | 2 |
| 130.3 | 157.3 | 0.4 | 0.1 | 1 | 25 | 1 |
| 129.4 | 156.0 | 0.4 | 0.1 | 1 | 15 | 2 |
| 125.6 | 149.3 | 0.4 | 0.1 | 1 | 15 | 1 |
| 136.4 | 166.0 | 0.5 | 0.1 | 2 | 25 | 1 |
| 139.3 | 166.7 | 0.63 | 0.1 | 1 | 15 | 2 |
| 130.9 | 161.3 | 0.35 | 0.1 | 2 | 25 | 2 |
| 132.9 | 157.5 | 0.55 | 0.1 | 1 | 15 | 1 |
| 133.2 | 160.2 | 0.5 | 0.1 | 1 | 15 | 2 |
