# Supplementary material for: 8.2% of the Human Genome Is Constrained: Variation in Rates of Turnover across Functional Element Classes in the Human Lineage
Source: PLoS Genet. 2014 Jul 24;10(7):e1004525. doi: 10.1371/journal.pgen.1004525 (PMC4109858; doi:10.1371/journal.pgen.1004525)
Supplement: Table S6 — The total quantities of constrained sequence estimated in the human genomes at present by different methods. The annotations are mutually exclusive sets as in Figure 4. (DOCX) [file pgen.1004525.s017.docx]

**Table S6: The total quantities of constrained sequence estimated in the human genomes at present by different methods.** The annotations are mutually exclusive sets as in Figure 4.

| **Annotations that extrapolations**  **are derived from** | **Percent of genome constrained at present (%; 95% confidence interval)** |
| --- | --- |
| Genome | 6.1 (5.3 – 7.0) |
| Coding, noncoding | 6.5 (5.4–7.6) |
| Coding, DNase HSs, TFBS, Enhancer, Promoter/UTR/lncRNA, Un-annotated | 8.2 (7.1 – 9.2) |
