# Supplementary material for: 8.2% of the Human Genome Is Constrained: Variation in Rates of Turnover across Functional Element Classes in the Human Lineage
Source: PLoS Genet. 2014 Jul 24;10(7):e1004525. doi: 10.1371/journal.pgen.1004525 (PMC4109858; doi:10.1371/journal.pgen.1004525)
Supplement: Text S1 — A new justification for the Neutral Indel Model 1 (NIM1). (DOCX) [file pgen.1004525.s018.docx]

**Text S1: A new justification for the Neutral Indel Model 1 (NIM1)**

The Neutral Indel Model (NIM1) estimates α_selIndel_ between a pair of species’ genome sequences. As described by Lunter *et al.* (2006) [[17](#_ENREF_1)], the model examines the distribution of intergap segments (IGSs) from a set of whole genome pairwise alignments using a regression approach over a range of medium IGS lengths to estimate the parameters of a predicted geometric distribution of IGSs in neutral sequence. α_selIndel_ in base pairs is then estimated as *x* - 2*K* bp summed over all the long IGSs inferred to be in excess above that predicted under neutral evolution, where *x* is the length of the overrepresented IGS, and *K* is the estimated mean spacing between indels in neutral sequence. 20 equally populated G+C content bins are each analysed separately, as is the X chromosome, to account, in part, for mutational variation.

We describe here two novel theoretical features of the model that were not previously identified in Lunter *et al.* (2006) [[17](#_ENREF_1)] or Meader *et al.* (2010) [[14](#_ENREF_2)]. These features are: (A) that thresholding can bias the expected lengths of the neutral overhang and, (B) that neutral segments are depleted from the background distribution due to the presence of constrained segments; we will now explain these features, and show that their contributions largely cancel out. The distance between successive indels (IGSs) in neutrally evolving sequence approximates a geometric distribution [[17](#_ENREF_1)]. Note that this holds true even in the presence of indel hotspots (SB Montgomery, DL Goode, E Kvikstad *et al.,* Genome Res. 2013; 23(5):749-61), provided that the hotspot locations are themselves generated by a uniform random process. Denote the mean IGS length in neutral sequence as *K*. The NIM1 consists of fitting a geometric model *h*(*x*) to the observed IGS histogram *H*(*x*), over the range of *x* that is dominated by neutral sequence; here *x* is the IGS length. Consider a functional segment consisting of *c* bases that does not accept indels. This segment contributes an IGS of expected length *c* + 2*K*, whose greater length is because of “neutral overhang” from either end of the segment to the nearest indel in neutral sequence; the expected distance to this first indel is *K* nucleotides on either side. The estimator α_selIndel_ for the total amount of constrained sequence is [*x* - 2*K*][*H*(*x*) – *h*(*x*)], summed over all *x*.

Feature (A) arises because in practice, in order to avoid the effects of gap attraction [[4](#_ENREF_4)], only contributions for *x* > *T* for some threshold *T* are included in this sum. This causes an upward bias to the expected neutral overhang, making the 2*K* correction too small, and resulting in an inflated estimate of α_selIndel_. Feature (B) arises because of the presence of constrained segments in the genome which skew the distribution of the remaining purely neutral segments. Imagine placing constrained segments randomly into an otherwise neutrally evolving genome with indels already located within them; because the segments are placed randomly, longer IGSs are more likely to be the recipients of such sequence than shorter ones, thereby depleting the set of neutral IGSs of longer segments. Since not only IGSs containing constrained sequence but also neutral IGSs contribute to *H*(*x*), this results in a lower estimate of α_selIndel_. The effect of (A) and (B) when taken together is that the estimator α_selIndel_ becomes conservative. To see this, consider an IGS of length *x* + *c* of which *c* nucleotides are conserved, assume that *h*(*x*) is correctly estimated, and that the threshold *T* ≤ 2*K*. Three cases are to be considered. (i) If *x* ≥ *T*, the segment contributes *x* + *c* – 2*K* to α_selIndel_. Due to effect (B) a segment *x* that would have contributed *x* – 2*K* is now missing from *H*(*x*), reducing α_selIndel_ by *x* – 2*K*. The total contribution is (*x* + *c* - 2*K*) - (*x* - 2*K*) = *c*, as desired. (ii) If *T* - *c* ≤ *x* < *T*, the segment *x* missed due to effect (B) is not included in the estimate (since *x* < *T*), so the contribution is *x* + *c* – 2*K* which is less than *c* since *x* < *T* ≤ 2*K*. (iii) If *x* < *T* - *c*, the segment makes no contribution. In no case is the contribution of the constrained segment overestimated, and hence α_selIndel_ underestimates the amount of constrained sequence.
