# Supplementary material for: 8.2% of the Human Genome Is Constrained: Variation in Rates of Turnover across Functional Element Classes in the Human Lineage
Source: PLoS Genet. 2014 Jul 24;10(7):e1004525. doi: 10.1371/journal.pgen.1004525 (PMC4109858; doi:10.1371/journal.pgen.1004525)
Supplement: Text S2 — Neutral Indel Model 2 (NIM2). (DOCX) [file pgen.1004525.s019.docx]

### Text S2: Neutral Indel Model 2 (NIM2)

We introduce an alternative Neutral Indel Model (NIM2) that estimates α_selIndel_ using a maximum likelihood approach. The model considers that indel mutations fall randomly across a wholly neutrally evolving genome into which conserved indel-free elements, representing functional sequence, are subsequently inserted. Although this construction does not reflect the actual series of evolutionary events, the resulting distribution of indels and conserved elements within neutral sequence is analogous, and the mathematical derivation is simplified. From this starting point, the distribution of medium-length and long IGSs is modeled, and α_selIndel_ is estimated by subtracting the cumulative length of the observed segments from the estimated length of the underlying neutral segments.

To account for gap attraction, the NIM2 describes the observed IGS counts above a lower length threshold *i*_min_. Its parameters are the total segment count *N*, the neutral average inter-gap distance *K*, and the per-base probability of insertion of functional sequence *p*. *N* is fixed to be the observed segment count, and as with NIM1 *i*_min_ and *i*_max_ are fixed describing the range [*i*_min_,*i*_max_] in which neutrally evolving sequence dominates, using the same range we used for NIM1.

Before introducing functional sequence, the expected number of IGSs of length *i* is $N_{i}=\frac{N}{K}{(1-\frac{1}{K})}^{i-i_{\text{min}}}$. An IGS of length *i* has probability *p*(*i*+1) of being the recipient of a functional segment, reducing the expected count to $\tilde{N}_{i}=\frac{N}{K}\left( 1-\frac{1}{K} \right)^{i-i_{\text{min}}}{(1-p)}^{i+1}$; later for technical convenience the approximation 1-*p*(*i*+1) ≈ (1-*p*)*^i^*^+1^ is used, valid as long as $p\ll\frac{1}{i}.$ The expected number of segments of length above *i*_max_, $\tilde{N}_{\text{above}},$consists of contributions from the $\tilde{N}_{i}$ in that range, and from neutral segments in the range [*i*_min_,*i*_max_] into which a functional segment was inserted. The observed counts are then modeled $N_{i}^{\text{obs}} (i\in[i_{\text{min}},i_{\text{max}}$]) and $N_{\text{above}}^{\text{obs}}=\sum_{i>i_{\text{max}}} N_{i}^{\text{obs}}$ as Bernoulli-distributed random variables with expectations $\tilde{N}_{i}$ and $\tilde{N}_{\text{above}}$; this choice does not enforce the sum of observed counts to equal *N*, but ignoring the small anti-correlation between observations that result from this constraint makes little difference in practice. Numerical methods implemented in R were used to infer maximum likelihood parameters for the given observed data. Finally, the inferred number of functional nucleotides was computed as the total number of nucleotides covered by observed IGSs, less the predicted total number of nucleotides covered by neutral segments *N_i_.*

Compared with the NIM1, the advantage of the NIM2 model is that features (A) and (B) are modeled explicitly. On the other hand, our implementation of NIM2 does assume a single genome-wide neutral indel rate, and thus does not account for known location-dependent mutational biases that NIM1 partially controls for by binning the genome based on G+C content and by analysing the X chromosome separately.
