# Supplementary material for: 8.2% of the Human Genome Is Constrained: Variation in Rates of Turnover across Functional Element Classes in the Human Lineage
Source: PLoS Genet. 2014 Jul 24;10(7):e1004525. doi: 10.1371/journal.pgen.1004525 (PMC4109858; doi:10.1371/journal.pgen.1004525)
Supplement: Text S3 — Alignment trimming improves alignment quality and αselIndel estimates. (DOCX) [file pgen.1004525.s020.docx]

**Text S3: Alignment trimming improves alignment quality and α_selIndel_ estimates**

We considered whether the manner in which the pairwise genome alignments were constructed influenced our previous estimates of α_selIndel_. To assess this we estimated α_selIndel_ with NIM1 and NIM2 on four different pairwise mouse-rat genome alignments produced by UCSC Genome Informatics using different alignment parameterisations and/or genome assemblies (Table S1). We used mouse-rat genome alignments since these are the most closely related species we can examine, and thus provide the most stringent test for the model due to their scarcity of indels. Estimated values of α_selIndel_ varied markedly depending on the assembly versions used and on the alignment parameters applied (Figure S1A, Figure S2, Table S1). To assess whether alignment quality influenced these results, we removed (‘trimmed’) poorly aligning sequence from these genome alignments, reasoning that such sequence is likely to be enriched with alignment and/or assembly errors. Rescoring and trimming of alignments using a log-odds approach (Materials and Methods) produced lower and more uniform NIM1 and NIM2 estimates across the various alignments (Figure S1A, Figure S2). Trimming removed substantial amounts of sequence that contained transposable elements and that were aligned with unexpectedly low sequence identity (Figure S1B, Table S2), and resulted in a sizeable reduction in the numbers of short IGSs (Figure S1C). These findings are compatible with an interpretation that trimmed sequence emanates from poor-quality non-orthologous alignments. Trimmed alignments were thus used for all subsequent analyses.

In further tests, we determined that there was only a small effect on estimates of α_selIndel_ when we removed either non-reciprocally aligning sequence between the two species or sequence that lay within known indel hotspots (SB Montgomery, DL Goode, E Kvikstad *et al.,* Genome Res. 2013; 23(5):749-61); consequently, we retained such sequences in subsequent analyses (Table S3).
