# Supplementary material for: 8.2% of the Human Genome Is Constrained: Variation in Rates of Turnover across Functional Element Classes in the Human Lineage
Source: PLoS Genet. 2014 Jul 24;10(7):e1004525. doi: 10.1371/journal.pgen.1004525 (PMC4109858; doi:10.1371/journal.pgen.1004525)
Supplement: Text S4 — Genome simulations demonstrate the accuracy and robustness of the NIMs. (DOCX) [file pgen.1004525.s021.docx]

## Text S4: Genome simulations demonstrate the accuracy and robustness of the NIMs

To test the performance of NIM1, we next set up an extensive series of simulations. While the model’s analysis shows that under the stated assumptions the original regression will provide unbiased results, we revisited other potential causes of bias that were not included in the model analysis by simulating data under a more detailed model and over a broader range of parameters than previously explored [14]. Specifically, we investigated the following parameters: species divergence, functional element clustering coefficient, indel fixation probability, size and shape parameters of the length distributions for both functional elements and intervening neutral sequences, and the residual indel rate variation; parameter definitions are provided in Table S4. We deemed it particularly important to test how robust the models are to variations in the residual indel rate because there is heterogeneity in the neutral indel rate that is not captured simply by partitioning the genome based on G+C content (SB Montgomery, DL Goode, E Kvikstad *et al.,* Genome Res. 2013; 23(5):749-61).

For each set of parameters, the evolution of two genomes sharing a common ancestral genome was simulated, with a constant 5% of the sequence evolving more slowly due to constraint (Figure 1A and Table S5). To reduce computation time, we simulated 200 Mb of sequence and scaled up the results to produce estimates for genomes of 3 Gb in size. Where possible, we estimated the parameter values required for the simulations from real data; when appropriate parameter values were difficult to obtain, a range of realistic values was used (Text S5). Following their simulated evolution, the descendant genomes were aligned using LASTZ (http://www.bx.psu.edu/miller_lab/), following UCSC’s alignment methodology, and the NIM1 was applied to determine how the inferred amount of constrained sequence compared to the known true amount.

For every one of 160 parameter combinations, the NIM1 α_selIndel_ estimate was conservative, but was never less than 80% of the true value (grey triangles in Figure 1A, Table S5). Importantly, the simulations also demonstrate that the model’s ability to accurately infer the amount of constrained sequence is not substantially diminished at the extremes of the divergence range (Figure 1A, Table S5). Consequently, we conclude that the NIM1 is robust: it does not overestimate α_selIndel_, even over the relatively small evolutionary distances we considered here. Furthermore, any trends in α_selIndel_ exceeding ~20% across the range of evolutionary distances cannot be attributed to any of the potential sources of bias we included in our simulations. Under the same simulations NIM2 produced relatively robust estimates of α_selIndel_, although it exhibited a slight diminution of power to detect constrained sequence with increasing divergence (Figure S3).
