# Supplementary material for: 8.2% of the Human Genome Is Constrained: Variation in Rates of Turnover across Functional Element Classes in the Human Lineage
Source: PLoS Genet. 2014 Jul 24;10(7):e1004525. doi: 10.1371/journal.pgen.1004525 (PMC4109858; doi:10.1371/journal.pgen.1004525)
Supplement: Text S5 — Simulating genome evolution. (DOCX) [file pgen.1004525.s022.docx]

**Text S5: Simulating genome evolution**

For each simulation, a 200Mb genome was simulated in 5kb blocks with G+C content distribution matching that of the human genome. 5% of this simulated genome was annotated as constrained, and was evolved at half the rate of the surrounding neutral sequence with respect to substitutions. This simulated genome was then used as ancestral sequence from which to evolve two descendant genomes at a specified divergence that represents the neutral substitution rate. Substitutions were modelled under the HKY85 model (with transition/transversion ratio of 2), and indel evolution was modelled by sampling indel lengths from a geometric distribution.

All relevant parameters are described in Table S4. The following parameters were fixed at a single value across all simulations. The functional length was fixed at 158.6bp, reflecting the mean length of GERP++ conserved elements [18]. The fixation probability was fixed at a value of 0.1, based on estimates from protein coding sequences (Brandstrom M, Ellegren H (2007), Genetics 176: 1691-1701). The Substitution/indel rate was fixed at 12, reflecting the values obtained across our alignments.

There is likely to be variation in the autosomal neutral indel rate due to mutational biases that are not accounted for by binning on G+C content. Consequently, we incorporated a degree of neutral indel rate heterogeneity into the model using a residual indel rate variation parameter (Table S4). To estimate the parameter value we first masked out all vertebrate PhastCons conserved elements from the trimmed hg19 – mm10 LASTZ alignment and then examined the distribution of indels in concatenated 5kb alignment blocks of the remaining putatively neutral sequence. We find that there is a mean and standard deviation of 164 and 20 indels per 5kb in these masked alignments. If there were to be no rate variation, and the indel frequencies were drawn from Poisson distributions, then the variance in the number of indels per 5kb would be 140 (i.e. a standard deviation of ~12). We observe a standard deviation of 20, i.e. a variance of 400, so an excess variance of 400-140 = 260. The variance of a uniform distribution across [x-L,x+L] is L^2^ / 3; setting this equal to 260 gives L=28, which translates to a value for the residual indel rate variation parameter of 28/140 = 0.2.
