# Supplementary material for: 8.2% of the Human Genome Is Constrained: Variation in Rates of Turnover across Functional Element Classes in the Human Lineage
Source: PLoS Genet. 2014 Jul 24;10(7):e1004525. doi: 10.1371/journal.pgen.1004525 (PMC4109858; doi:10.1371/journal.pgen.1004525)
Supplement: Text S6 — Technical artefacts cannot explain observed signatures of turnover. (DOCX) [file pgen.1004525.s023.docx]

## Text S6: Technical artefacts cannot explain observed signatures of turnover

## We observe a strong negative correlation between estimates of α_selIndel_ and the divergence of the two species compared in the alignment (Figure 1A), consistent with substantial turnover of functional sequence and thus with earlier conclusions [14,16], and inconsistent with simulation results under a scenario in which turnover is absent. However, it is *a priori* conceivable that these observations are nevertheless the result of an unspecified technical artefact that we failed to consider, leading to a lack of specificity when identifying constrained sequence in particular for less divergent species pairs. We sought to exclude this possibility, by showing that lineage-specific constrained sequence identified by NIM1 is enriched for sequence with biochemical annotation for function. In addition we exclude the possibility of indel rate variation coinciding with ENCODE functional annotations driving the observations.

To argue the first point, let us hypothesize that functional sequence does *not* exhibit turnover to any significant degree. We will show that this hypothesis is incompatible with observations. Invoking the premise that function equates to present-day constraint, sequence with associated experimental evidence for biochemical function in human (“putative functional sequence”) but that also lacks evidence for long-term, pan-mammalian conservation, would represent either (1) a false positive of the functional experiment and in fact be neutrally evolving sequence, or (2) a false negative of the algorithms to detect pan-mammalian conservation and actually be pan-mammalian but weakly conserved, functional sequence. (The third possibility, short-lived constrained sequence that was not identified by algorithms not optimized for this type of constraint, is excluded by hypothesis.) In the first case, we do not expect such neutral sequence to be enriched among purportedly constrained sequence identified by the NIM1, as their distinguishing feature (the ability to cause a false positive in a functional experiment) is not expected to correlate with a spurious lack of indels in neutral sequence (we specifically address, below, the logical possibility of lower indel rate in regions annotated by ENCODE as functional, which could then potentially drive this enrichment). In the second case, one would not expect the NIM1, which uses only the signal of indels between pairs of species, to detect such pan-mammalian but weakly conserved sequence with better power than algorithms that integrate the signal of single-nucleotide substitutions across multiple species, and that were specifically designed to detect the signal of pan-mammalian conservation. Either way, under the assumption that functional sequence exhibits turnover to a minimal degree, we conclude that putative functional sequence that shows no signature of pan-mammalian constraint would not be enriched with NIM1-constrained sequence.

Instead, we observe precisely this enrichment, as we now discuss. We used two substitution-based methods that identify pan-mammalian conserved sequence: the two-state phylogenetic HMM PhastCons [11], and the position specific model GERP++ [18] (Cooper GM, Stone EA, Asimenos G, Green ED, Batzoglou S, et al. (2005) Genome Res 15: 901-913). Between closely related species, NIM1 identifies substantial amounts (e.g. 24 Mb between human and horse) of putatively constrained sequence that are not detected by either PhastCons or GERP++ as being pan-mammalian conserved (Figure S4). As putatively lineage-specific functional sequence we took mutually exclusive sets of predicted enhancers (530 Mb), transcription factor binding sites (TFBSs; 79 Mb), and DNase 1 hypersensitive sites (DNase HSs; 116 Mb) as defined from experimental evidence by the ENCODE project [4], that in addition do not overlap either PhastCons or GERP++ conserved elements. Within each of these sets, NIM1 identifies significantly higher fractions of constrained sequence compared to a control set of sequence (p=8×10^-8^, p=1×10^-4^, p=2×10^-10^; Figure 2A; Figure 3C). The control set was defined as unannotated sequence lacking experimental evidence for function, not covered by PhastCons or GERP++ elements, not within 50 bp of enhancers, DNAse HSs, TFBSs, or promoters as defined by ENCODE, not within 50 bp of protein coding exons or UTRs as defined by Ensembl, and not within 50 bp of the lncRNAs from [[11](#_ENREF_11)] (1148 Mb in total for this control set).

In summary, the observation of significant enrichment of NIM1-constrained sequence within putatively functional sequence that does not show evidence for pan-mammalian constraint is incompatible with the hypothesis that functional sequence does not exhibit turnover to a significant degree. We therefore conclude that the signature of functional turnover identified by NIM1 (Figure 1A) is not driven by its lack of specificity for low species divergence, but instead indicates that turnover of functional sequence is pervasive.

Second, we address the possibility of unaccounted-for variation in indel rates correlating with ENCODE-annotated functional regions driving the signal. Specifically, if neutral indel rates were systematically lower in ENCODE functional regions, for reasons other than GC content which we correct for, the NIM1 may over-estimate the amount of constrained sequence in such regions, leading to the observed signal. If neutral indel rates were indeed reduced in ENCODE functional regions, low-frequency polymorphic indels should be depleted from such regions. To test this we looked at the incidence of 1000 Genomes indels with derived allele frequency <2% in the CEU (Utah individuals with Northern and Western European ancestry) and YRI (Yoruba individuals from Ibadan, Nigeria) populations (SB Montgomery, DL Goode, E Kvikstad *et al.,* Genome Res. 2013; 23(5):749-61). We only used indels that could be reliably polarized so that the DAF could be calculated. For CEU we find 15.3±0.3, 15.2±0.5 and 14.9±0.6 indels per Mb in the enhancer, TFBS and DNAse HS categories, and 15.0±0.2 / Mb in unannotated sequence (errors are 2 standard deviations under a Poisson count model). For YRI the numbers are 27.6±0.4, 26.9±0.6, 26.6±0.9 indels/Mb respectively, and 26.3±0.2 indels/Mb in unannotated sequence. The only significantly different pair of estimates are indel rates in enhancers and unconstrained sequence measured using YRI indels, and these data point to a somewhat higher (by ~5%) indel rate in enhancers sequence compared to unannotated sequence, contrary to what would be expected if lower indel rates were driving the observations.

For completeness, we show more directly that NIM1 is highly specific also for divergent species pairs. Over larger evolutionary distances, virtually all sequence identified as being constrained by NIM1 is also found by PhastCons and GERP++; for example, 97% of NIM1 constrained sequence is identified as being conserved by PhastCons and/or GERP++ between the relatively divergent genomes of human and mouse.
