# Supplementary material for: 8.2% of the Human Genome Is Constrained: Variation in Rates of Turnover across Functional Element Classes in the Human Lineage
Source: PLoS Genet. 2014 Jul 24;10(7):e1004525. doi: 10.1371/journal.pgen.1004525 (PMC4109858; doi:10.1371/journal.pgen.1004525)
Supplement: Text S7 — Modelling turnover of pan-mammalian conserved sequence. (DOCX) [file pgen.1004525.s024.docx]

## Text S7: Modelling turnover of pan-mammalian conserved sequence

To assess whether putatively functional sequence lacking evidence for pan-mammalian conservation, more specifically sequence not covered by PhastCons [11] or GERP++ [18] conserved elements, shows enrichment for NIM1-inferred conservation at the present day (data presented in Figure 2), it might seem that we can use the same approach to infer the amount of present-day constraint elements in particular annotation classes, namely apply NIM1 to the genomic sequence minus the PhastCons/GERP++ elements and fit the results to the turnover model $\alpha=ae^{-bd}$. However this would be incorrect because we have purposely removed sequence showing evidence for pan-mammalian conservation, resulting in a reduction of $\alpha$ independent of *d*. This could be modelled by introducing an additional offset parameter to the model, but we found that this caused the model to be over-determined because of the relatively few data points that were available. Instead, as an alternative we fit to a linear model $\alpha=a-bd$; since the turnover model is concave, this leads to conservative estimates for the *a* parameter under the hypothesis of turnover described by the model. To be consistent we used the same model for the null; since there is no expectation as to the dependence of the observations of artefactual constraint $\alpha$ on the divergence under the null hypothesis, a linear model appears a robust choice.
