# Supplementary material for: 8.2% of the Human Genome Is Constrained: Variation in Rates of Turnover across Functional Element Classes in the Human Lineage
Source: PLoS Genet. 2014 Jul 24;10(7):e1004525. doi: 10.1371/journal.pgen.1004525 (PMC4109858; doi:10.1371/journal.pgen.1004525)
Supplement: Text S8 — Levels of sequence constraint for protein coding sequences. (DOCX) [file pgen.1004525.s025.docx]

**Text S8: Levels of sequence constraint for protein coding sequences**

80-88% of human and 80-86% of mouse defined protein coding sequences are estimated be under selective constraint by the NIM1. Lower proportions (68–71%) of dog annotated coding sequence are inferred to have been under constraint (Figure S6), likely reflecting the lower abundance of transcriptional evidence and thus the less refined gene annotations for the dog genome (Derrien T, Vaysse A, Andre C, Hitte C (2012) Mamm Genome 23: 124-131). While NIM2 shows qualitatively the same trends, its estimates of protein coding constraint under this model are somewhat lower (72–81% for the human or mouse genome, 64% for the dog genome). Results from application of the two models are consistent with the accepted notion that most protein coding gene sequence is highly conserved (Nielsen R, Hellmann I, Hubisz M, Bustamante C, Clark AG (2007) Nat Rev Genet 8: 857-868). Since NIM1 consistently shows greater sensitivity in identifying protein coding sequence than NIM2 (Figure S6), we focused on NIM1 estimates for all subsequent analyses.
