# Supplementary material for: 8.2% of the Human Genome Is Constrained: Variation in Rates of Turnover across Functional Element Classes in the Human Lineage
Source: PLoS Genet. 2014 Jul 24;10(7):e1004525. doi: 10.1371/journal.pgen.1004525 (PMC4109858; doi:10.1371/journal.pgen.1004525)
Supplement: Supplementary file 16 [file pgen.1004525.s005.docx]

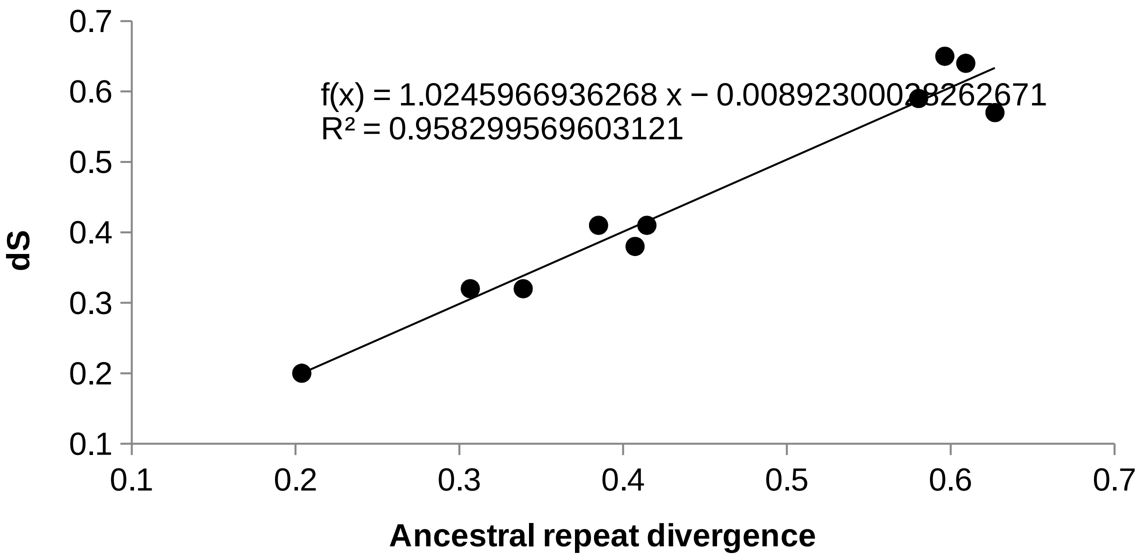

f(x) = 1.0245966936268 x − 0.00892300028262671
R² = 0.958299569603121
dS
0.7
0.6
0.5
0.4
0.3
0.2
0.1
0.1
0.2
0.3
0.4
0.5
0.6
0.7
Ancestral repeat divergence
