# Supplementary material for: 8.2% of the Human Genome Is Constrained: Variation in Rates of Turnover across Functional Element Classes in the Human Lineage
Source: PLoS Genet. 2014 Jul 24;10(7):e1004525. doi: 10.1371/journal.pgen.1004525 (PMC4109858; doi:10.1371/journal.pgen.1004525)
Supplement: Supplementary file 17 [file pgen.1004525.s006.docx]

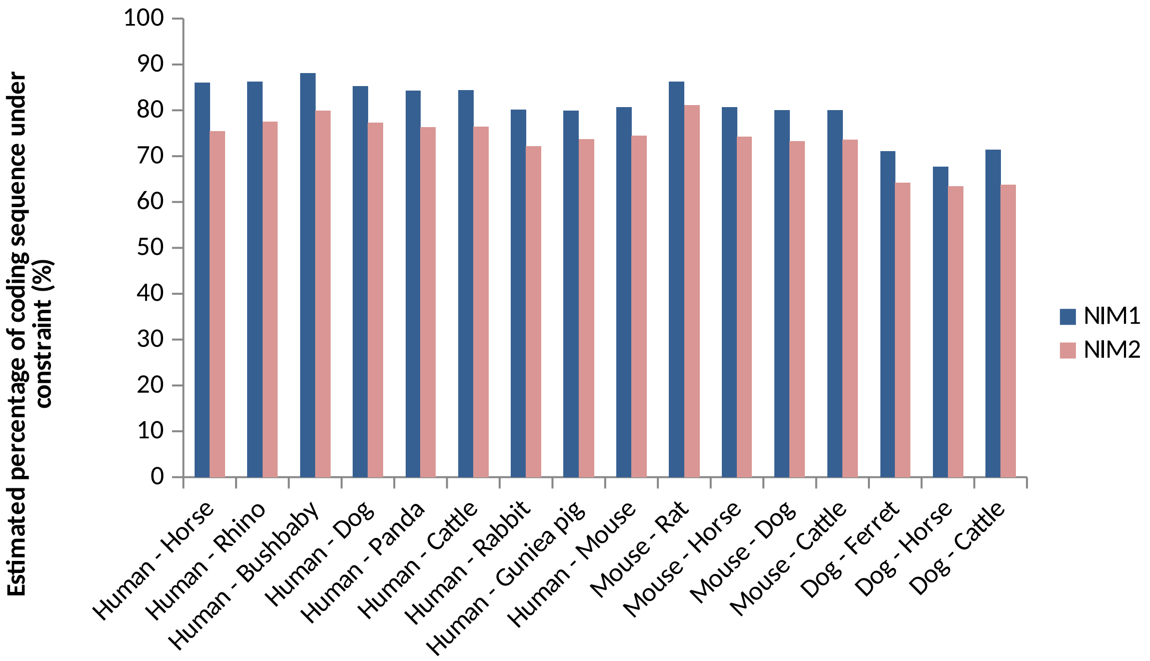
Estimated percentage of coding sequence under constraint (%)
100
90
80
70
60
50
40
30
20
10
0
Human - Horse
Human - Rhino
Human - Bushbaby
Human - Dog
Human - Panda
Human - Cattle
Human - Rabbit
Human - Guniea pig
Human - Mouse
Mouse - Rat
Mouse - Horse
Mouse - Dog
Mouse - Cattle
Dog - Ferret
Dog - Horse
Dog - Cattle
NIM1
NIM2
