# Supplementary material for: 8.2% of the Human Genome Is Constrained: Variation in Rates of Turnover across Functional Element Classes in the Human Lineage
Source: PLoS Genet. 2014 Jul 24;10(7):e1004525. doi: 10.1371/journal.pgen.1004525 (PMC4109858; doi:10.1371/journal.pgen.1004525)
Supplement: Supplementary file 18 [file pgen.1004525.s011.docx]

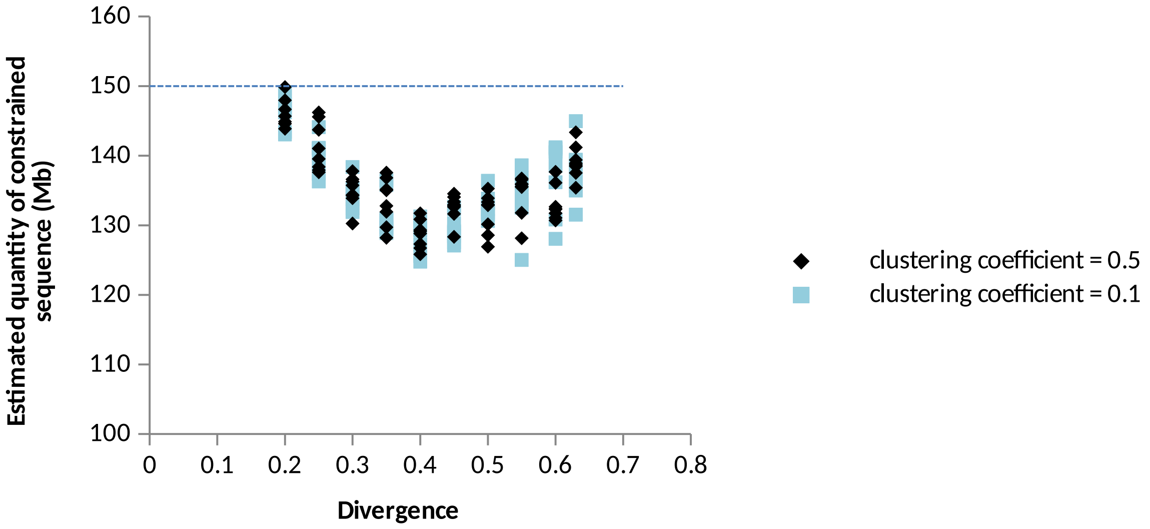
Estimated quantity of constrained sequence (Mb)
160
150
140
130
120
110
100
0
0.1
0.2
0.3
0.4
0.5
0.6
0.7
0.8
Divergence
clustering coefficient = 0.5
clustering coefficient = 0.1
